# The anterior Hox gene *ceh-13* and *elt-1/GATA* activate the posterior Hox genes *nob-1* and *php-3* to specify posterior lineages in the *C. elegans* embryo

**DOI:** 10.1371/journal.pgen.1010187

**Published:** 2022-05-02

**Authors:** John Isaac Murray, Elicia Preston, Jeremy P. Crawford, Jonathan D. Rumley, Prativa Amom, Breana D. Anderson, Priya Sivaramakrishnan, Shaili D. Patel, Barrington Alexander Bennett, Teddy D. Lavon, Erin Hsiao, Felicia Peng, Amanda L. Zacharias

**Affiliations:** 1 Department of Genetics, Perelman School of Medicine, University of Pennsylvania, Philadelphia, Pennsylvania, United States of America; 2 Division of Developmental Biology, Cincinnati Children’s Hospital Medical Center, Cincinnati, Ohio, United States of America; 3 Department of Pediatrics, University of Cincinnati College of Medicine, Cincinnati, Ohio, United States of America; NYU School of Medicine, UNITED STATES

## Abstract

Hox transcription factors play a conserved role in specifying positional identity during animal development, with posterior Hox genes typically repressing the expression of more anterior Hox genes. Here, we dissect the regulation of the posterior Hox genes *nob-1* and *php-3* in the nematode *C*. *elegans*. We show that *nob-1* and *php-3* are co-expressed in gastrulation-stage embryos in cells that previously expressed the anterior Hox gene *ceh-13*. This expression is controlled by several partially redundant transcriptional enhancers. These enhancers act in a *ceh-13*-dependant manner, providing a striking example of an anterior Hox gene positively regulating a posterior Hox gene. Several other regulators also act positively through *nob-1/php-3* enhancers, including *elt-1/GATA*, *ceh-20/ceh-40/Pbx*, *unc-62/Meis*, *pop-1/TCF*, *ceh-36/Otx*, and *unc-30/Pitx*. We identified defects in both cell position and cell division patterns in *ceh-13* and *nob-1;php-3* mutants, suggesting that these factors regulate lineage identity in addition to positional identity. Together, our results highlight the complexity and flexibility of Hox gene regulation and function and the ability of developmental transcription factors to regulate different targets in different stages of development.

## Author summary

Hox genes are critical for head-to-tail patterning during embryonic development in all animals. Here we examine the factors that are necessary to turn on two posterior Hox genes, *nob-1* and *php-3*, in the nematode worm, *C*. *elegans*. We identified six new transcription factors and three enhancer regions of DNA that can activate expression of *nob-1/php-3*. Unexpectedly, these activating transcription factor genes included *ceh-13*, an anterior Hox gene, and *elt-1*, a regulator of skin development that is briefly expressed in many cells that do not adopt skin fates, including the cells that express *nob-1*. Furthermore, the cellular defects we observed in *ceh-13* and *nob-1;php-3* mutant embryos indicate that the early embryonic functions of these Hox genes help determine the identity of cells as well as their position within the embryo. Our findings identify new roles for Hox genes in *C*. *elegans* and emphasize the ability of transcription factors to contribute to the diversification of cell types and the adoption of specific cell types at different phases of embryonic development.

## Introduction

Hox genes encode conserved transcription factors famously expressed in specific positions along the anterior-posterior axis during animal development that specify axial position. Mutations in Hox genes cause a wide variety of developmental defects in both model organisms and humans. Hox gene regulation is complex and includes both transcriptional and post-transcriptional control. In most animals, Hox genes are found in genomic clusters, and their expression along the A-P axis is collinear with their genomic position, and often show “posterior dominance,” where posterior Hox genes repress the expression of more anterior Hox genes (Reviewed in [1–3]).

The genome of the nematode *Caenorhabditis elegans* encodes a single set of six Hox genes on Chromosome III, loosely organized into three degenerate “clusters” that each contain two adjacent genes [3–6]. The cluster containing *lin-39* and *ceh-13* is inverted relative to Hox clusters in other organisms as *ceh-13*, a homolog of *HoxA1/labial*, is downstream of *lin-39*, a homolog of *HoxA4/Deformed* [7–9]. In the most distal cluster, *php-3* lies 220 bp directly downstream of the 3’UTR of *nob-1* and while large deletions in either gene are viable, disrupting both loci or the likely *cis-*regulatory regions upstream of *nob-1* results in embryonic lethality, suggesting these genes are redundant and co-regulated during embryonic development [5,10]. In larval stages, these genes are expressed in specific positions along the A-P axis and regulate both positional differences in cell fate and function, similar to their homologs in other animals [11–19], and directly regulate terminal fates [10,20].

Three *C*. *elegans* Hox genes, the anterior Hox gene homolog *ceh-13/HOX1* and the posterior Hox gene homologs *nob-1/HOX9-13* and *php-3/HOX9-13*, are also expressed in early embryogenesis, during gastrulation [5,21–23]. *ceh-13* mutants have severe defects in anterior (head) morphology [22], while mutants lacking both *nob-1* and *php-3* have severe posterior (tail) defects [5]. Partial cell lineage tracing of *ceh-13* and *nob-1*;*php-3* mutants identified defects in cell position but not in division patterns, leading to the hypothesis that these genes regulate positional identity, rather than lineage identity [5,6,22].

Intriguingly, despite their apparently opposite roles in anterior vs posterior morphogenesis, both *ceh-13* and *nob-1* are expressed in overlapping posterior lineages during gastrulation and both require the Wnt pathway for this expression [24,25]. *ceh-13* is transiently expressed in the progeny of 7 of the 8 posterior sister cells derived from the (largely ectoderm-producing) AB blastomere at the 24-cell stage [21]. *nob-1* is expressed 1–2 cell cycles later, in the posterior daughters or grand-daughters of four of these *ceh-13*-expressing cells [23]. In addition, both *ceh-13* and *nob-1* are expressed in the posterior daughter of the intestinal blastomere E (“Ep”). This raises the question of whether *ceh-13* regulates *nob-1* in these lineages. Expression of both factors at later stages is regulated by feedback mechanisms; early *ceh-13* activity is required for later *ceh-13* expression [25], and early *nob-1* negatively regulates later *nob-1* expression through the microRNA *mir-57* [23].

Here we analyzed the *cis-*regulatory control of *nob-1/php-3* expression in the early embryo. We find that *nob-1/php-3* expression is regulated by several partially redundant distal enhancers, including at least three that drive overlapping patterns during gastrulation. We find that *ceh-13* is required for normal timing and levels of *nob-1/php-3* expression, by activating at least two of its enhancers. We further identified the GATA family transcription factor gene *elt-1*, previously known as a specifier of epidermal fate, as one of several additional positive regulators of *nob-1/php-3* expression. Detailed analysis of cell positions and cell division timing identifies both position defects and defects in cell division patterns and timing in *ceh-13* and *nob-1*/*php-3* mutants. This role in cell identity combined with the positive regulation of a posterior Hox gene by an anterior Hox gene suggest novel roles for Hox genes in early lineage specification.

## Results

### The anterior Hox gene *ceh-13* and the posterior Hox gene *nob-1* are expressed sequentially in gastrulating embryos

**Fig 1:**
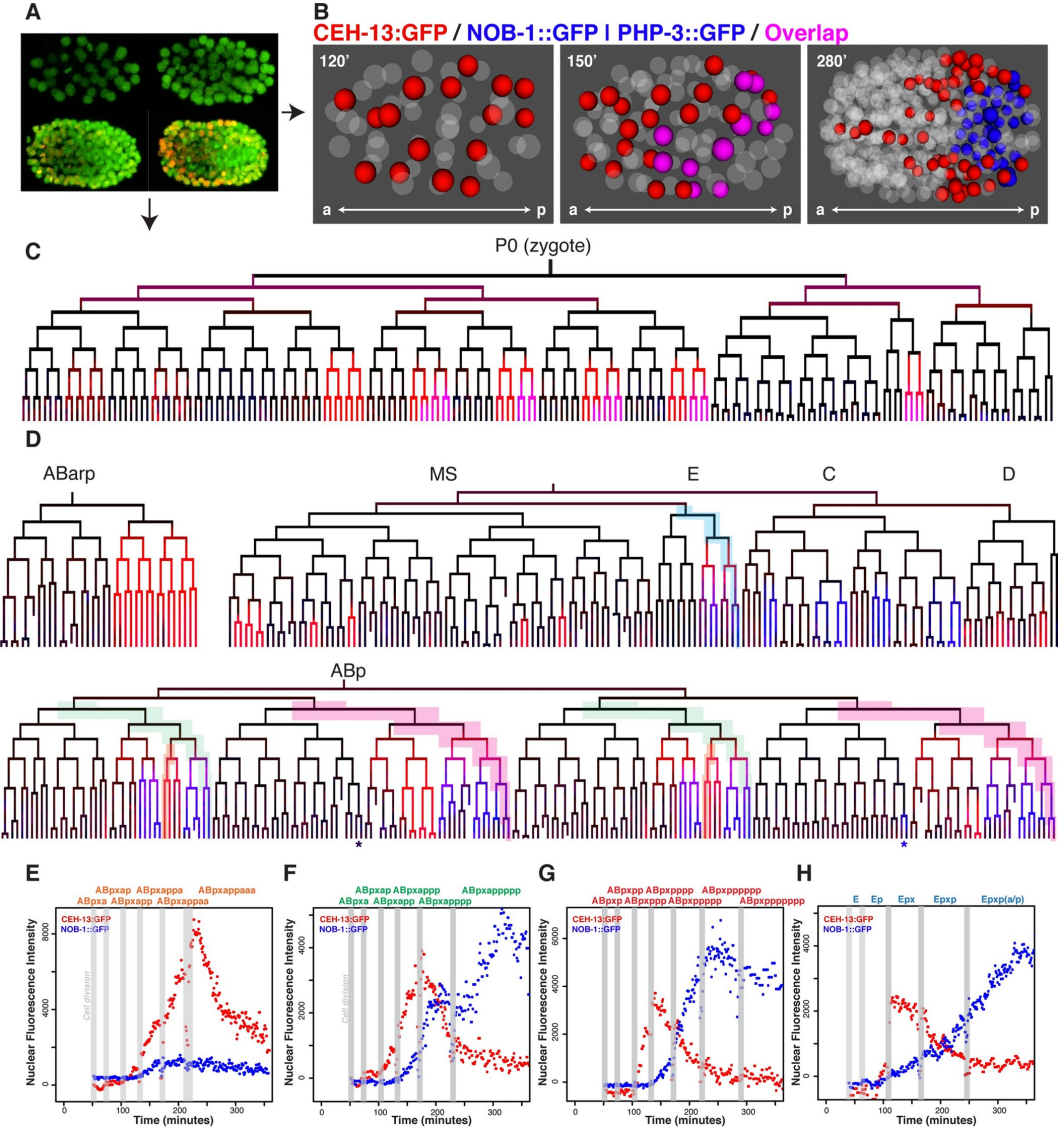
*ceh-13* and *nob-1/php-3* are expressed broadly in an overlapping pattern in the early *C*. *elegans* embryo. A) Time-lapse images of transgenic *C*. *elegans* embryos carrying two transgenes, a ubiquitous fluorescent histone to mark all nuclei (shown in green) and a reporter of interest (shown in red), which can be a cis-regulatory element driving a fluorescent histone (transcriptional) or a fluorescently tagged transcription factor protein (translational). Image analysis software identifies nuclei, and quantifies reporter intensity within the nuclei, which can be displayed as a lineage tree colored by expression as in (C). B) 3D projections of nuclei expressing endogenously tagged CEH-13::GFP (red) and endogenously tagged NOB-1::GFP and PHP-3::GFP (blue), with overlap shown in magenta, at 120 minutes (50 cell stage), 150 minutes (100 cell stage), and 280 minutes (400 cell stage) post fertilization. Endogenously tagged NOB-1::GFP and PHP-3::GFP were expressed in indistinguishable patterns (S1 Fig), but PHP-3::GFP was slightly brighter so is shown in the following panels. C) Lineage tree through the 100-cell stage, showing early expression of endogenously tagged CEH-13::GFP and PHP-3::GFP, colored as in (B). D) Expressing lineages showing endogenously tagged CEH-13::GFP and/or PHP-3::GFP expression to 350 minutes of development, colors as in (B). Note that CEH-13::GFP precedes PHP-3::GFP and NOB-1::GFP in all lineages except ABp(l/r)papppp (asterisk—expression is consistent in ABprpapppp and variable in ABplpapppp). (E-H) Quantitative detail for highlighted lineages, showing nuclear fluorescence intensity of CEH-13::GFP (fosmid) and NOB-1::GFP transgene reporters across developmental time for the cells leading to ABp(l/r)appaaa (E), ABp(l/r)appppp (F), ABp(l/r)ppppppp (G), and Ep(l/r)p (H). For cell labels, x = (l/r). Nuclear fluorescence intensity is in arbitrary units. Grey bars mark cell divisions.

To better understand the expression dynamics of the *ceh-13*, *nob-1*, and *php-3* Hox genes during embryogenesis (Fig 1), we collected 3D time-lapse movies (~1.5 minute temporal resolution) of embryos expressing either *ceh-13*, *nob-1* or *php-3* modified at the endogenous locus by CRISPR/Cas9 genome editing to tag the encoded proteins with green fluorescent protein (GFP) at the C-termini. The same embryos also expressed a ubiquitously expressed mCherry-tagged histone transgene to allow for cell lineage tracing. We traced cells from soon after fertilization (4–8 cell stage) through the last round of cell divisions for most cells (bean stage) by using StarryNite automated cell tracking software, and quantified reporter expression in each cell across time [26–29]. Note, each endogenously tagged Hox::GFP fusion line is homozygous viable, fertile, and displays no obvious phenotypes, demonstrating that the fusion proteins largely function like the wild-type proteins.

Consistent with previous studies using shorter reporters [21], endogenously tagged CEH-13::GFP expression is first seen in eight Wnt-signaled cells (that are the more-posterior daughter after cell division) and their progeny at the 26-cell stage, during early gastrulation (Figs 1B, 1C, and S2). These include the AB lineage-derived cells ABalap, ABalpp, ABarpp, ABplap, ABplpp, ABprap and ABprpp, and the posterior endoderm progenitor Ep. For simplicity, we use the term “lineage” to denote all cells derived from a specific blastomere; thus, the “Ep lineage” would refer to all descendants of Ep. The ABalap and ABalpp lineage expression was barely detectable and quickly faded, while the other lineages had more robust and persistent fluorescence, indicating the protein was degraded or maintained in distinct cells. By the 200–350 cell stage, many of the granddaughters of the initially expressing cells have lost CEH-13::GFP expression, while a few cells fated to become blast cells or neurons sustained or increased their expression. Also at this stage, some cells in the D and MS lineages as well as a few additional AB-derived cells show CEH-13::GFP accumulation (Fig 1D). We compared the tagged protein expression pattern to that of endogenous mRNA as measured in a lineage-resolved single cell RNA-seq dataset, and found that the mRNA and tagged protein expression patterns were consistent [30]. In comparison, a rescuing 35 kb CEH-13::GFP fosmid transgene had brighter expression in the cells expressing the endogenously tagged protein, plus additional weak expression detectable in some epidermal precursors from the C lineage (possibly detectable due to higher copy number). A shorter reporter containing only 8.2 kb of upstream sequence lacked expression in the MS lineage, emphasizing the role of distal regulatory elements in regulating *C*. *elegans* Hox gene expression (S1D Fig) [31,32].

Next, we defined the embryonic expression patterns of the posterior Hox genes *php-3* and *nob-1* tagged with GFP at the endogenous locus. As expected, *nob-1* and *php-3*, which are adjacent in the genome with *php-3* directly downstream of *nob-1*, are expressed in identical patterns in both our GFP imaging data (S1A Fig) and in the single-cell RNA-seq data [30]. Expression of both NOB-1::GFP and PHP-3::GFP begins during mid-gastrulation in cells derived from the cells ABplapp, ABplppp, ABprapp, ABprppp, Ep, and the four posterior great-granddaughters of the C blastomere. Expression becomes stronger one cell cycle later in the AB-derived lineages with the exception of that of ABp(l/r)appa. Additional late embryonic expression occurs in the ABp(l/r)papppp lineages starting at the onset of morphogenesis (“bean” stage). NOB-1-expressing lineages give rise to fates including neurons, hypodermis, seam cells, epithelial cells, intestine, and death, and have posterior positions clustered in and near the developing tail at comma stage [33]. Like CEH-13, early NOB-1::GFP expression persists through the terminal cell divisions in only a subset of cells (Fig 1D). A previously described NOB-1::GFP rescuing transgene that includes 9kb of sequence upstream of the *nob-1* transcript expresses GFP in a similar pattern, except it is not expressed in the C great-granddaughter lineages, suggesting this expression requires additional regulatory sequences outside of this region (Figs 1D and S1) [23].

Early CEH-13::GFP expression occurs in cells that are distributed broadly along the anterior-posterior axis (Fig 1B), while endogenously tagged NOB-1::GFP and PHP-3::GFP expression is more limited to the posterior of the embryo. In the AB lineage, NOB-1/PHP-3 are expressed exclusively in cells that were the posterior sister after cell division and whose mother expressed CEH-13 (Fig 1D–1H). In addition, these three genes are co-expressed at similar onset times in the posterior intestine lineage derived from Ep. In contrast, in late embryos, high levels of CEH-13::GFP and NOB-1::GFP/PHP-3::GFP are largely mutually exclusive (Figs 1D, S1F, and S1G), consistent with classic models of Hox expression and posterior dominance.

### Several overlapping lineage specific enhancers regulate *nob-1* embryonic expression

**Fig 2:**
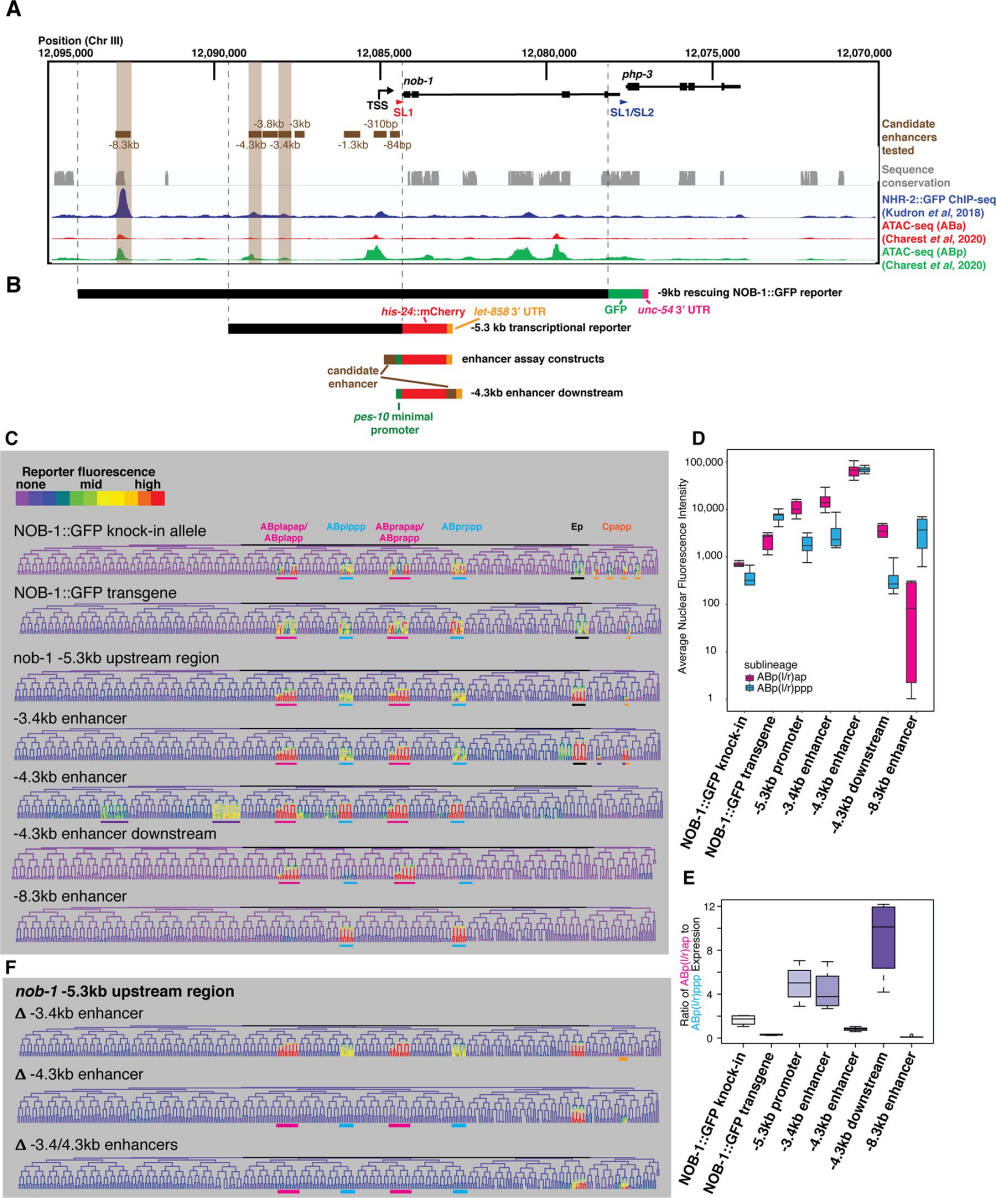
Regions upstream of *nob-1/php-3* can recapitulate its expression pattern. A) Genome browser view of the *nob-1*/*php-3* locus showing the genes (black), candidate enhancers tested (brown), sequence conservation with other nematodes (grey), and NHR-2 ChIP-seq trace (dark blue) from modENCODE [43] and ATAC-seq traces (red:ABa lineage, green: ABp lineage) from Charest et al, 2020 [66]. B) Schematics of the *nob-1* reporter constructs examined, shown in alignment with (A). Enhancers were primarily tested in an orientation 5’ to the *pes-10* minimal promoter, but a downstream orientation was also used for the -4.3 kb enhancer. C) Lineage trees colored to show expression patterns for the various reporters and tested enhancers with relevant reproducible activity, using a rainbow color scale to increase visible dynamic range. Major expressing lineages are underlined: ABp(l/r)ap: pink, ABp(l/r)ppp: cyan, Ep: black, Cpapp: orange, ectopic: purple. Note the changes in expression driven by the -4.3 kb enhancer depending on its position relative to the *pes-10* minimal promoter. D) Average nuclear fluorescence values for cells in the ABp(l/r)ap (pink) and ABp(l/r)ppp (blue) lineages from at least four embryos, shown on a log scale (arbitrary units). E) Ratio of expression in average ABp(l/r)ap cell to the average ABp(l/r)ppp cell at the 350 cell stage for at least 4 embryos (n = 4–7). F) Lineage trees showing expression driven by the nob-1–5.3 kb upstream region reporter with the -3.4 kb and/or -4.3 kb enhancers are deleted. Lineages where expression is lost are underlined with colors as in (C).

To identify regulators of *nob-1* embryonic expression, we tested nearby genomic sequences for embryonic *cis-*regulatory activity (Fig 2A and 2B). Previous work showed that other *C*. *elegans* Hox genes are regulated by distal enhancers located as much as 20kb from a given gene’s promoter [25,31,34], but enhancers for *nob-1* and *php-3* have not been identified. We took advantage of existing reporters of different lengths to narrow the sequence search space for *nob-1* embryonic enhancers (Fig 2C). A transcriptional reporter containing just 5.3 kb of upstream sequence driving histone-mCherry reporter expression, and the rescuing 9kb NOB-1::GFP reporter are expressed in most of the same lineages as endogenous NOB-1::GFP, indicating they contain regulatory sequences sufficient for expression in the full set of expressing lineages. However, while the protein fusion reporters are expressed at similar levels in each lineage, the shorter 5.3kb transcriptional reporter drives much lower fluorescence in the ABp(l/r)ppp lineages compared to other lineages (Fig 2C and 2D). This suggests that additional sequences between -5.3kb and -9kb are required for the full endogenous expression levels in the ABp(l/r)ppp lineage.

Although other *Caenorhabditis* species have large (12-20kb) intergenic regions upstream of *nob-1*, there is no detectable sequence conservation in the 5.3kb region upstream of *nob-1*, and only two short conserved stretches (85 and 275 nucleotides long) between 5.3 and 9kb upstream [35–38]. This indicates that any conserved regulatory elements in this region have diverged substantially at the sequence level during *Caenorhabditis* evolution, limiting utility of evolutionary conservation to identify enhancers. Chromatin marks typically used to identify transcriptional enhancers such as H3K27ac, H3K4me, or chromatin accessibility from whole embryos did not show strong peaks near *nob-1* or near 20 additional genes expressed in lineage-specific patterns at the same stage as *nob-1* [32,39–41].

We hypothesized that these chromatin signals are diminished in early embryonically expressed genes because the experiments used embryos of mixed stages where later stage nuclei dramatically outnumber nuclei from earlier stages. Therefore, we searched for other factors with published genomic binding patterns that could mark embryonic enhancers. One pattern stood out as preferentially bound near genes expressed with lineage-specific patterns in the early embryo: binding of a NHR-2::GFP fusion protein [42,43]. NHR-2::GFP binds in the intergenic sequences upstream of 19 of these 20 genes, and 16 genes have multiple clustered NHR-2 binding sites (vs. 22% and 8% of randomly chosen genes, respectively, p < 0.001; chi-squared test). NHR-2 is a nuclear hormone receptor distantly related to mammalian thyroid and PPAR receptors, and NHR-2::GFP is expressed in most or all somatic cells from the ~50-cell to ~200-cell stages. The functional importance of NHR-2 binding is unclear; *nhr-2* RNAi causes embryonic and larval arrest, but partial deletion alleles are viable. However, regardless of its function, we hypothesized that these clustered NHR-2 binding sites might be useful proxies for accessible chromatin and could predict enhancer activity in the early embryo.

We tested four NHR-2::GFP-bound regions upstream of *nob-1* for enhancer activity by generating transgenic worms expressing histone-mCherry under the control of each candidate enhancer placed upstream of a *pes-10* minimal promoter, which drives no consistent embryonic expression on its own (Fig 2A and 2B). We also tested four additional sequences for which we observed no NHR-2::GFP binding but which contained putative binding motifs for POP-1/TCF, which is required for expression of the *nob-1* transcriptional reporter [24]. We identified embryonic cells expressing each enhancer reporter by confocal time-lapse imaging and StarryNite. Only three of the regions (located at -3.4 kb, -4.3 kb and -8.3 kb) tested showed enhancer activity that was consistent between embryos and also overlapped with the endogenous NOB-1::GFP expression pattern, suggesting they represent functional enhancers (Figs 2C and S2). All three functional enhancers were identified on the basis of NHR-2 binding.

The enhancer located at -3.4 kb (hereafter referred to as the -3.4kb enhancer) recapitulates most of the *nob-1* early embryonic expression pattern, with some differences in the C lineage, and drives much weaker expression in ABp(l/r)ppp than in ABp(l/r)app, similar to the *nob-1*–5.3 kb upstream transcriptional reporter. The enhancer located at -4.3 kb also drives expression in ABp(l/r)app and ABp(l/r)ppp at a very high level (Fig 2D) as well as variable misexpression in cells that do not normally express NOB-1 (Figs 2C and S2A). When this enhancer was cloned downstream of the reporter, it produced less ectopic expression and stronger expression in ABp(l/r)app relative to ABp(l/r)ppp, similar to the -3.4kb enhancer. This confirms the -4.3 kb region contains a *bona fide* enhancer capable of acting at a distance and suggests that the placement of this enhancer relative to the promoter influences its activity differently in different lineages and may be important for specificity. Embryos carrying multiple copies of the -4.3 kb reporter transgene occasionally displayed the “no backend” phenotype observed in *nob-1/php-3* mutant embryos (S2B Fig) [5]. A third *nob-1* enhancer (located at -8.3 kb) drives early embryonic expression only in ABp(l/r)ppp, as well as later expression in ABp(l/r)papppp. This most-distal enhancer is included in -9 kb NOB-1::GFP transgene but not the shorter -5.3kb transcriptional reporter, and includes the only substantial stretch of conserved sequence in the *nob-1* promoter region. This region thus can explain why ABp(l/r)ppp lineage expression is relatively stronger in the translational reporter compared to the shorter transcriptional reporter.

As the -5.3kb *nob-1* transcriptional reporter includes both the -3.4kb and -4.3kb enhancers, we deleted each enhancer from this construct to test their necessity for *nob-1* expression (Fig 2C). Deleting both enhancers led to a complete loss of early embryonic expression in both AB lineages, suggesting there are no additional enhancers present sufficient for early embryonic expression in these lineages. The deletion of the -3.4kb enhancer did not disrupt reporter fluorescence in the ABp(l/r)app and ABp(l/r)ppp lineages, and in the absence of the other enhancer located at -4.3 kb, this enhancer was not sufficient to drive reporter fluorescence in these lineages. This indicates that while the -3.4kb enhancer may be sufficient to drive expression when placed directly next to the promoter, it is unable to drive expression in the endogenous context. Conversely, the -4.3kb enhancer is a key driver of expression in these lineages. The reporter fluorescence that remains when both enhancers are absent indicates that additional sequences that drive the expression in the cells of the Ep and Cpapp lineages must exist within the -5.3 kb upstream region.

### *ceh-13* and Hox cofactor genes activate *nob-1* expression

**Fig 3:**
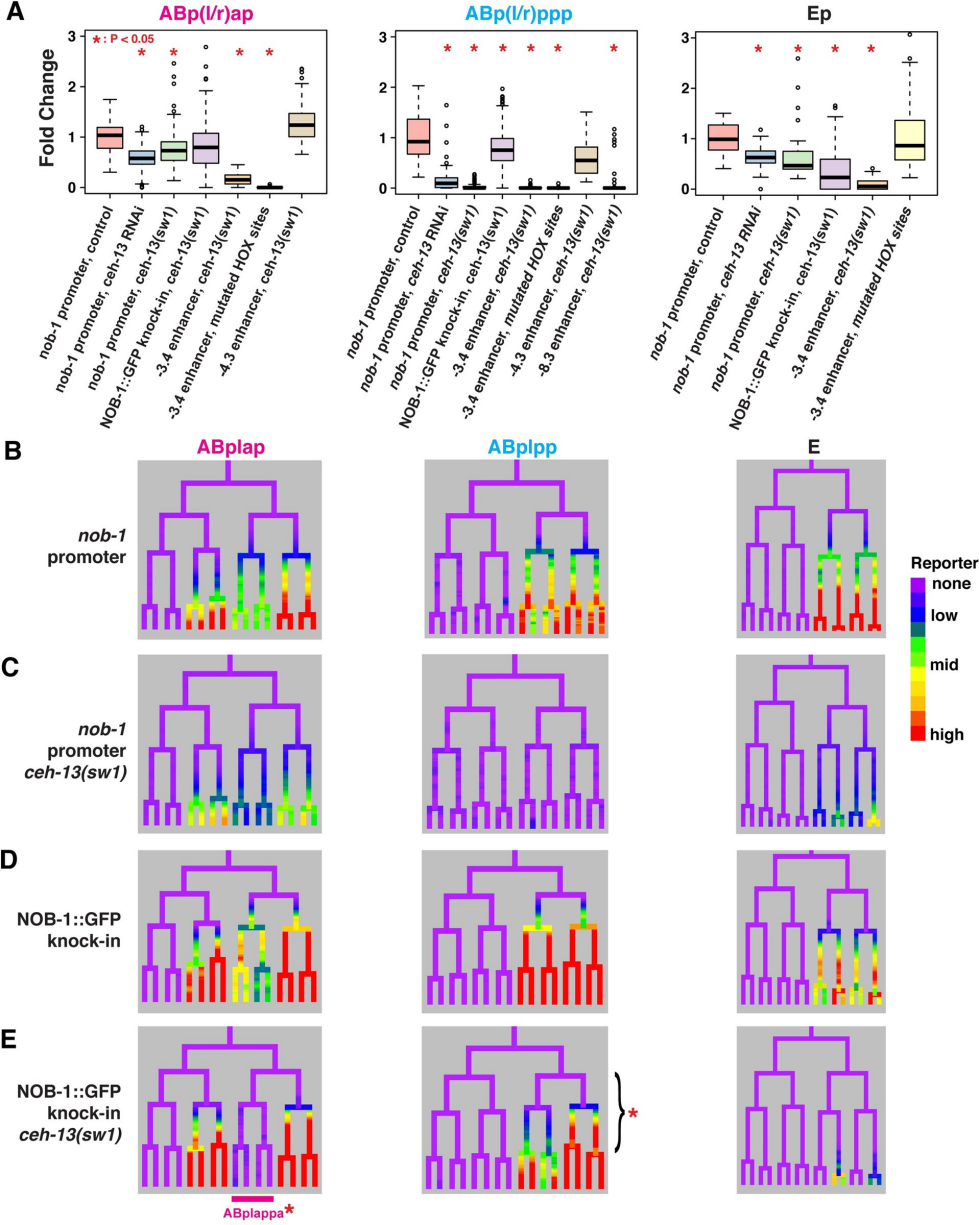
Expression of cis-regulatory elements upstream of *nob-1* and *php-3* depends on *ceh-13*. A) Fold change in expression level relative to mean wild-type control for *nob-1* promoter, endogenously tagged NOB-1::GFP knock-in allele, and enhancer reporters in *ceh-13* mutant and RNAi conditions. Number of biological replicates for each condition ranges from 3–16, however, since there are, for example, 36 *nob-1* cells in the ABp(l/r)ap lineages, the number of points represented by the box plots is much greater. *P* values determined by Wilcoxon Ranked Sum Test, p<0.05 significant. B) Lineage view of wild-type *nob-1*–5.3kb upstream reporter (“promoter”) expression in specified lineages. C) -5.3kb *nob-1* promoter reporter expression in *ceh-13(sw1)* null mutant. D) Lineage view of wild-type endogenously tagged NOB-1::GFP expression in specified lineages. E) Expression of endogenously tagged NOB-1::GFP in *ceh-13(sw1)* null mutant. Underlined lineages and bracketed early cells had specifically significantly lower NOB-1::GFP levels.

Because CEH-13::GFP expression precedes that of NOB-1::GFP in many lineages, we asked whether *ceh-13* is required for *nob-1* expression. To evaluate this, we examined *nob-1* reporter fluorescence in embryos homozygous for the likely null mutation *ceh-13(sw1)* [22]. Fluorescence driven by the -5.3kb *nob-1* transcriptional reporter is absent in the ABp(l/r)ppp lineage (p < 0.001), 25% lower in the Abp(l/r)ap lineage, and 40% lower in the Ep lineage (p < 0.02) (Fig 3A–3C). Depletion of *ceh-13* by RNAi gave similar results with the transcriptional reporter (p < 0.002). To test the relevance of this to the endogenous locus, we measured fluorescence levels of endogenously tagged NOB-1::GFP knock-in allele in *ceh-13(sw1)* mutant embryos. This fluorescence was reduced in the Ep, Abp(l/r)appa, and Abp(l/r)ppp lineages by 59%, 49%, and 23% respectively (P < 0.001) (Fig 3A, 3D, and 3E). The initial onset of expression of NOB-1::GFP was delayed relative to control embryos in many of these cells (Figs 3E and S3B). Expression of endogenously tagged PHP-3::GFP in *ceh-13(sw1)* mutant embryos showed similar changes to endogenously tagged NOB-1::GFP (S3C Fig), indicating both genes are regulated by *ceh-13*, possibly through the same enhancers. In addition, the fact that the *nob-1* promoter reporter completely loses ABp(l/r)pp lineage expression in the absence of *ceh-13* while the endogenously tagged alleles show only temporal delay and quantitative reduction indicates the importance of additional *cis-*regulatory sequences outside of the promoter region for *nob-1* and *php-3* expression.

To determine which enhancers require *ceh-13*, we measured the activity of individual *nob-1* enhancer reporters in worms lacking *ceh-13* (Figs 3A and S3A). In *ceh-13(sw1)* mutants, reporter fluorescence driven by the -3.4 kb enhancer decreased significantly in all expressing cells, excluding the C lineage (p < 0.003) with an average reduction of 90% and complete absence of reporter fluorescence in ABp(l/r)ppp. *ceh-13* loss also reduces reporter fluorescence driven by the -4.3 kb enhancer in ABp(l/r)ppp (47% decrease) but fluorescence in Abp(l/r)ap is unchanged. This indicates that other factors besides *ceh-13* activate the -4.3kb enhancer in the Abp(l/r)ap lineage and suggests that this enhancer may be responsible for the residual expression of the 5.3 kb transcriptional reporter in this lineage in *ceh-13* mutant embryos. Fluorescence from the transgene driven by the distal -8.3kb enhancer was also absent in ABp(l/r)ppp in the *ceh-13(sw1)* mutant (p < 0.002), indicating its activity also requires *ceh-13*. Motif analysis identified six putative *ceh-13*-binding motifs in the -3.4 kb enhancer; mutating these sites resulted in a lack of reporter fluorescence in the AB and C lineages (p < 0.02, p < 0.03), but did not affect reporter fluorescence in the Ep lineage indicating *ceh-13* regulation of *nob-1* may be indirect in the E lineage (Figs 3A and S3B). These results show that multiple *ceh-13*-dependent enhancers work together to regulate *nob-1/php-3* expression in gastrulating embryos.

**Fig 4:**
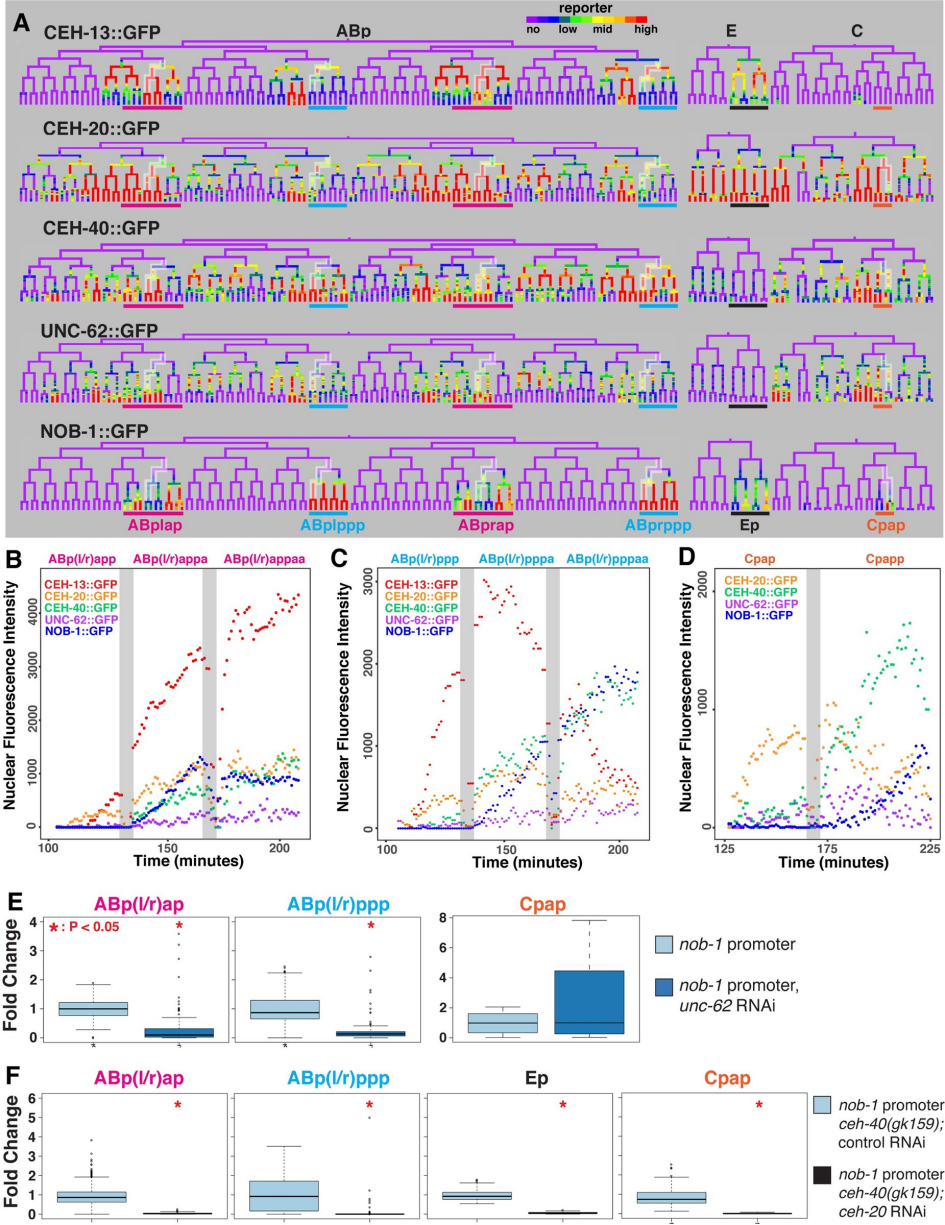
Hox co-factors precede *nob-1* and regulate its expression. A) Trees of NOB-1::GFP expressing lineages, ABp, E, and C, showing the expression of *ceh-13* (fosmid) and *nob-1* (GFP transgene) reporters, and fosmid GFP transgene reporters for the Hox co-factor genes, *ceh-20*, *ceh-40* and *unc-62*. Color thresholds were adjusted for each reporter to show all expressing cells. Highlighted branches are shown in graphs B, C, and D. B-D) Average TF reporter nuclear fluorescence intensity across embryos (n ≥ 2) and for left/right symmetric cells across developmental time for the cells leading to B) ABp(l/r)appaa, C) ABp(l/r)pppaa and D) Cpapp. Fluorescence intensity is in arbitrary units and grey bars mark cell divisions. E,F) Fold change values for -5.3 kb *nob-1* promoter expression in untreated (n = 5) and *unc-62* RNAi treated (n = 7) (E) or *ceh-40(gk159)* mutant embryos treated with *ges-1* (control, n = 5) or *ceh-20* RNAi (n = 5) (F) in specified lineages. Significant changes (p<0.05) marked by asterisk; *P* values determined by Wilcoxon Ranked Sum Test.

Expression of classically defined Hox targets often requires Hox cofactors such as those encoded by *homothorax* or *extradenticle*. These cofactors form larger TF complexes with Hox factors to increase binding specificity and may also have Hox-independent functions [44–46]. To determine when and where Hox cofactors are expressed in early embryos, we used StarryNite to trace the expression of translational reporters for *unc-62*, the *C*. *elegans homothorax/Meis* ortholog, and of *ceh-20* and *ceh-40*, the orthologs of *extradenticle/Pbx*. A third *extradenticle* ortholog, *ceh-60*, is only expressed in later development (>200 minutes) and does not overlap with *ceh-13*, so was not investigated further [30]. We found that fosmid translational reporter transgenes for each of these genes show specific and dynamic expression patterns that overlap with each other and with *ceh-13* and *nob-1* expression (Figs 4A–4D and S5). Notably, all three cofactors are co-expressed with CEH-13 in AB-derived cells that will later express NOB-1. Other CEH-13 and NOB-1 expressing cells also express all three cofactors, except only CEH-20 is expressed in the early E lineage, and all three are lost in the NOB-1-expressing ABp(l/r)appp lineage as the embryo approaches morphogenesis. We conclude that Hox cofactors are expressed in cells where *ceh-13* activates *nob-1* expression.

We tested whether Hox cofactors regulate *nob-1* expression by examining *nob-1* reporter expression after loss of each gene. We found that depleting *unc-62* by RNAi led to significantly less expression of the *nob-1*–5.3kb reporter in all AB lineages (80% reduction, p = 0.001), with near complete absence of reporter fluorescence in the ABp(l/r)apap and ABp(l/r)ppp lineages (Fig 4E). The -9kb NOB-1:GFP protein reporter transgene showed similarly limited expression, although this was only significant in the ABp(l/r)appa lineage and variable in other lineages (S4B and S4C Fig). In contrast, CEH-13::GFP reporter expression was unchanged after *unc-62* RNAi (S4D Fig). These results show that *nob-1* is regulated by *unc-62*. The fact that *unc-62* RNAi has a stronger phenotype in the ABp(l/r)apap lineage than loss of *ceh-13* suggests that *unc-62* may regulate *nob-1* independently of *ceh-13* in these cells.

To investigate the role of the *extradenticle* orthologs, we examined the *nob-1* transcriptional reporter in a *ceh-40(gk159)* null mutant background in embryos of worms treated with control or *ceh-20* RNAi. We found that the combination of *ceh-20* RNAi with the *ceh-40* mutation resulted in an absence of reporter fluorescence from virtually all expressing cells including the Ep and Cpap lineages as compared to control RNAi (97% decrease, p < 0.004). This indicates that the *exd* orthologs *ceh-20* and *ceh-40* are required to activate regulatory elements upstream of *nob-1* and that they are at least partially independent of *ceh-13* in the cells of the ABp(l/r)ap, Ep, and Cpap lineages. This finding is consistent with previous reports that *nob-1* RNAi causes increased lethality in a *ceh-40* mutant background relative to wild-type animals [47].

### Multiple genes encoding lineage-specific embryonic TFs are required for early *nob-1* expression, including *elt-1/GATA*, *ceh-36/OTX* and *unc-30/PITX*

Since some NOB-1::GFP expression remains in the absence of *ceh-13*, especially in the ABp(l/r)ap lineage, other factors must also activate *nob-1* in these cells. Indeed, we previously identified the Wnt effectors *pop-1* and *sys-1* as activators of *nob-1* expression [24]. We took advantage of databases of TF expression to search for additional candidate regulators [30,41,48].

**Fig 5:**
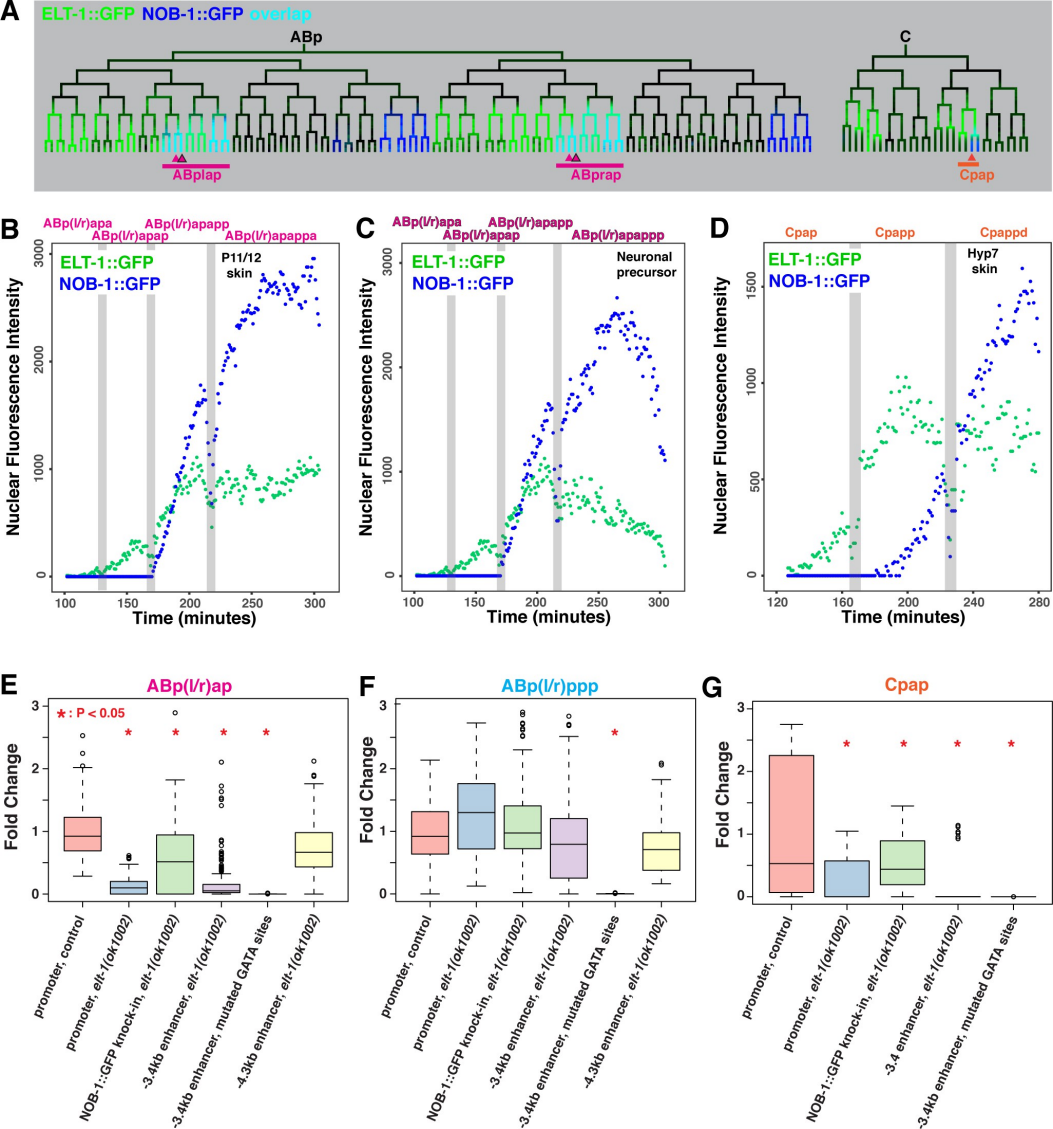
*elt-1* regulates *nob-1* expression in ABp(l/r)ap lineage. A) Partial lineage trees showing transgene expression patterns of ELT-1::GFP (green), NOB-1::GFP (blue) or overlapping (Cyan) expression in the ABp and C lineages. Arrowheads indicate branches shown in subsequent panels. B-D) Average transgenic ELT-1::GFP and NOB-1::GFP nuclear fluorescence intensity (arbitrary units) in cells leading to B) ABp(l/r)apappa (P11/12 blast, part of the ventral hypodermis in the embryo) C), ABp(l/r)apappp (neuroblast fate), and D) Cpappd (hypodermal fate). Grey bars mark cell divisions. E-G) Fold change values for the *nob-1* promoter, endogenously tagged NOB-1::GFP, and -3.4 kb and -4.3 kb wild-type or mutated enhancer reporters in the ABp(l/r)ap lineage in the control and *elt-1(ok1002)* mutant conditions in the E) ABp(l/r)ap, F) ABp(l/r)ppp, and G) Cpap lineages. Number of biological replicates ranges from 4–6. Significant changes (p<0.05) marked by red asterisk; *P* values determined by Wilcoxon Ranked Sum Test.

Only two of the three enhancers (-3.4kb and -4.3kb) drive expression in the ABp(l/r)ap lineage, so to identify additional potential regulators of *nob-1* in this lineage, we used two criteria. First, we searched for TFs expressed specifically in ABp(l/r)ap, but not ABp(l/r)pp. Second, we used a TF binding site scanning approach to identify which of these TFs have predicted binding motifs in the -3.4kb and -4.3kb enhancers [9,49]. From this analysis, the GATA zinc finger TF encoded by *elt-1* emerged as the strongest candidate. An ELT-1::GFP reporter is observed prior to gastrulation in the ABp(l/r)ap progenitors ABpla and ABpra, in the other major epidermis-producing lineages ABarp, Caa and Cpa, and in the primarily neuronal lineages ABalap and ABalppp (Fig 5A). ELT-1::GFP fluorescence is later downregulated in cells that do not adopt epidermal fates (Fig 5C). ELT-1::GFP fluorescence in the ABpla/ABpra lineages could be detected at least 20 minutes prior to *nob-1* reporter expression in ABp(l/r)ap (Fig 5B and 5C). GATA motifs predicted to bind ELT-1 are enriched in the enhancers that are active in ABp(l/r)ap, -3.4kb (8 sites) and -4.3kb (7 sites), compared to the -8.3kb enhancer (1 site), which does not drive expression in ABp(l/r)ap. While the *C*. *elegans* genome encodes several other GATA factors, only *elt-1* is expressed prior to *nob-1* in the ABp(l/r)a lineage [30,50].

To test whether *elt-1* regulates *nob-1* expression, we measured the fluorescence of *nob-1* reporters in the *elt-1(ok1002)* null mutant background (Figs 5E–5G, S6A, and S6B). We found that *elt-1* mutants showed a complete loss of endogenously tagged NOB-1::GFP expression in the ABp(l/r)apap lineage (p<0.0002) and reduced expression in the ABp(l/r)appa (40% reduction, p<0.002), Caap and Cpap (35% reduction, p<0.02) lineages where ELT-1::GFP precedes NOB-1::GFP expression (Figs 5E–5G and S6A–S6C). Furthermore, we observed lower reporter fluorescence from the *nob-1* transcriptional reporter in the ABp(l/r)ap lineage (87% reduction, p < 0.014), and the Cpap lineage (92% reduction, not significant), but not in ABp(l/r)pp, where ELT-1::GFP is not expressed (Fig 5F). Similarly, we observed less reporter fluorescence in the same lineages for the -3.4kb enhancer reporter (ABp(l/r)ap: 72% reduction, p < 0.008; C: 71% reduction, p < 0.03) and the -4.3kb enhancer reporter, particularly in ABp(l/r)apap (73% reduction, p < 0.02) (Figs 5E–5G and S6A). To determine if *elt-1* directly regulates the -3.4kb enhancer, we mutated the predicted ELT-1 binding sites, which have a characteristic GATA motif, and observed an absence of reporter fluorescence in all lineages (p < 0.001). We conclude that *elt-1* is required to activate *nob-1* expression in the ABp(l/r)ap lineage through at least two enhancers, and is at least partially redundant with other factors that may bind GATA motifs.

We previously showed that two homeodomain *bicoid* family TF genes, *ceh-36/OTX* and *unc-30/PITX*, are redundantly required for proper development of the ABp(l/r)p lineage [51]. Since *ceh-13* and *elt-1* mutants still have some remaining NOB-1::GFP expression in the ABp(l/r)p descendants, we hypothesized that *ceh-36* and *unc-30* might be important for *nob-1* expression in these lineages. All three enhancers contain at least two putative CEH-36/UNC-30 binding sites, and the -8.3 kb enhancer, which activates reporter fluorescence only in ABp(l/r)p, has nine (Tables C-E in S1 Table). To test this hypothesis, we examined expression of the NOB-1::GFP transgene (which includes all three enhancers) in eight *ceh-36;unc-30* double mutant embryos, and found that two of the eight embryos (25%) lacked reporter fluorescence in this lineage (S7A Fig). Furthermore, the ratio of ABp(l/r)ap to ABp(l/r)ppp reporter fluorescence significantly increased (p < 0.002) in the remaining double mutant embryos, consistent with a decrease in ABp(l/r)ppp expression (S7B Fig). In conclusion, at least seven partially redundant lineage-specific transcription factor genes positively regulate *nob-1* expression: *ceh-13*, *ceh-20*, *ceh-40*, *unc-62*, *elt-1*, *ceh-36*, and *unc-30*.

### ceh-13 and nob-1 are required for correct cell position and division patterns in the early embryo

**Fig 6:**
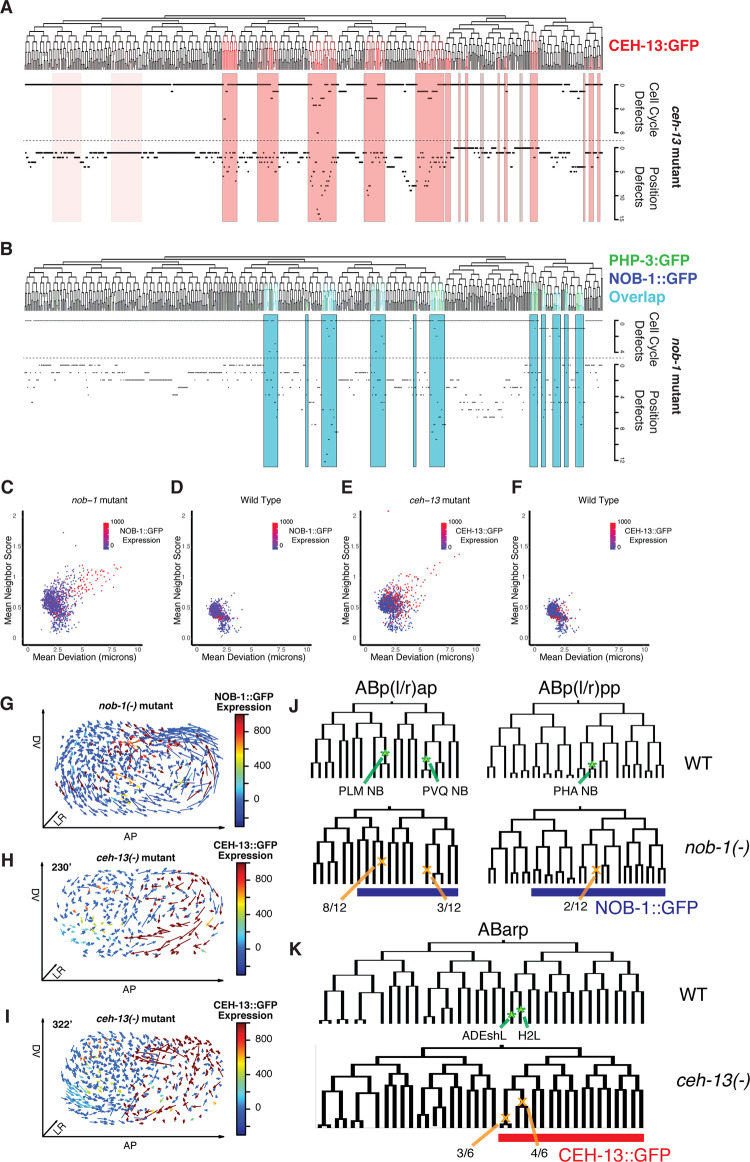
Cell defects are observed in the posterior of both *ceh-13* and *nob-1* mutant embryos. A) Lineage showing CEH-13::GFP expression with plots of frequency of cell cycle defects and cell position defects for each cell plotted below. Cells expressing CEH-13::GFP during their development are highlighted in pink (ABalap and ABalpp, which express very low levels of CEH-13::GFP, are denoted with lighter shading). Cell cycle defects are defined as missed, ectopic or significant change in division timing of at least 5 minutes and cell position defects are defined as significant deviation of at least 5 μm from the positions expected from wild-type (see methods). Defects are listed cumulatively for terminal cells, so defects in both a parent and daughter cell would be scored as two defects. B) Lineage showing NOB-1::GFP and PHP-3::GFP expression overlap with plots of frequency of cell cycle defects and cell position defects for each cell plotted below. Cells expressing both NOB-1::GFP and PHP-3::GFP are highlighted in cyan on the plots. C, D) Plot of mean deviation in microns vs. mean neighbor score for each cell in the embryo, colored by level of NOB-1::GFP expression, for *nob-1(ct223)* mutant which disrupts both *nob-1* and *php-3* (C) and wild-type (D) embryos. Neighbor score is a measure of whether a cell is close to its normal neighbors; values above 0.8 indicate aberrant neighbor relationships. E, F) Plot of mean deviation in microns vs. mean neighbor score for each cell in the embryo, colored by level of CEH-13::GFP expression, for *ceh-13(sw1)* mutant (E) and wild-type (F) embryos. (G-I) Three-dimensional plot of cell position deviations—arrows point from average wild-type location to average mutant location for each cell, colors indicate the level of NOB-1::GFP (G) or CEH-13::GFP(H-I) expression: G) *nob-1(ct223)* at 335 minutes of development. H) *ceh-13(sw1)* at 230 minutes of development) *ceh-13(sw1)* at 322 minutes of development. J) Wild-type and *nob-1(ct223)* mutant lineages for the ABp(l/r)ap and ABp(l/r)pp lineages, showing examples of cell division defects. Green stars indicate the normal divisions of neuroblasts, orange Xs indicate the failed divisions of these cells with the frequency observed noted below. Blue underline indicates the expression of NOB-1::GFP. K) Wild-type and *ceh-13(sw1)* mutant lineages for the ABarp lineage. Green stars indicate the normal development of ADEshL and H2L cells, orange Xs indicate the ectopic division of these cells with the frequency observed noted below. Red underline indicates the expression of CEH-13::GFP.

Previous work suggested that *nob-1/php-3* and *ceh-13* are required for cell position but not for other aspects of cell fate specification [5]. However, mutating lineage identity regulators often leads to defects in cell cycle length or division patterns [51,52] that could have been missed in previous studies. To test for such defects, we measured cell positions and cell division timing in six embryos carrying the *nob-1(ct223)* mutation, which also deletes *php-3* (referred to as *nob-1* mutant for simplicity), and *ceh-13(sw1)* mutant embryos by time-lapse microscopy and StarryNite cell tracking and compared them to a database of 17 wild-type embryos (Fig 6) [28,46,51].

Consistent with previous work [5], we observed striking global patterns of cell position defects in *nob-1* mutant embryos. The most severe and highest penetrance defects were in cells from NOB-1-expressing lineages (Figs 6A, 6C, 6D, 6G, and S8). In total, 48% (62 of 127) of cells descended from NOB-1::GFP expressing lineages had severe position defects (>5 micron deviation, z score > 3.5) in at least two embryos compared to 1.2% (14 of 1094) of cells from non-expressing lineages (p <<10^−20^). However, a large number of cells from non-expressing lineages were moderately mis-positioned, suggesting that many defects are not cell-autonomous. Globally, dorsal cells that normally express NOB-1 fail to migrate to the posterior at 230 minutes post-fertilization; by 300 minutes non-expressing ventral cells compensate by inappropriately moving to the posterior, resulting in a global counterclockwise rotation of cell positions when the embryo is viewed from the left aspect (Fig 6G). The position defects we observed are consistent with the severe posterior morphology defects observed in rare surviving larvae.

We also identified broad cell position defects in *ceh-13* mutant embryos (Fig 6B, 6E, 6F, 6H, and 6I). Intriguingly, the global patterns of cell position defects differed from *nob-1* mutants. The number of severe position defects was lower for *ceh-13* than for *nob-1*, with 4% (15/357) of cells descended from CEH-13::GFP expressing lineages severely displaced in at least two embryos, as opposed to 0.5% (5/874) of cells from non-expressing lineages (p << 10^−20^). At 210 minutes, (200 cell stage), the most strongly mispositioned cells were *ceh-13-*expressing cells that do not express *nob-1* in the posterior ventral region of the embryo. At this stage, cells on the dorsal surface were posteriorly mispositioned, such that cells in the posterior half of the embryo were rotationally mispositioned clockwise, the opposite direction to that seen in *nob-1* mutants (Fig 6H). Cells at the anterior ventral surface (both *ceh-13*-expressing and non-expressing) were mispositioned towards the posterior, resulting in a counterclockwise rotational defect in the anterior half of the embryo. At later stages, these global defects were largely resolved, and after the onset of morphogenesis the position defects were largely limited to a small number of strongly mispositioned cells (Fig 6I). The most extreme were the DA and SAB motor neurons after 300 minutes (bean stage), which were mispositioned several cell diameters anterior of their wild-type position (S8B Fig). Given the recovery of most cell positions by the beginning of morphogenesis, it is unclear if cell position defects contribute to the severe anterior morphogenesis defects in *ceh-13* mutants that hatch [22], although the earlier cell position defects could disrupt normal cell-cell signaling interactions required for fate specification.

In contrast to previous studies [5,22], we also identified numerous defects in cell division timing or pattern in both *nob-1* and *ceh-13* mutants, and these were also heavily restricted to expressing lineages (Fig 6A and 6B). 13% of *nob-1* expressing cells and 5% of *ceh-13* expressing cells had severe division-time defects in at least two of the corresponding mutant embryos (>5 minute deviation from wild-type, and z-score >5), vs. none in non-expressing cells (p << 10^−20^). The most common cell division defect in *nob-1* mutants was the PLM/ALN neuroblast mother (ABp(l/r)apappp), which failed to divide in 8 of 12 observations (Fig 6J). The neuroblast that normally produces the PHA, PVC, and LUA neurons instead underwent programmed cell death in two of 12 mutant lineages (Fig 6J). Other cells that had multiple cell cycle delays in *nob-1* mutants included the PVQ neuroblast, PHB/HSN neuroblast, and the mother of the epidermal cells PHsh, hyp8 and hyp9 (Fig 6J and Table J in S1 Table). All of these cells also were mispositioned (mean 5 micron deviation from expected position, S8C Fig). The most striking defects in *ceh-13* mutants were in the cells ABarppaaa(a/p), which normally differentiate into the ADE sheath (glial) cell and H2 epidermal cell, but instead each divided inappropriately in 4 of 6 mutant embryos (Fig 6K). In addition, ABplppaaap, which normally undergoes programmed cell death, instead divided in two *ceh-13* mutant embryos (Table I in S1 Table). Several neuroblasts including those producing the DA, DD and SAB motorneurons also had cell division delays in *ceh-13* mutants (Table I in S1 Table). We confirmed that the most severe cell division defects have similar penetrance in additional alleles of *ceh-13* and *nob-1/php-3* (S8D Fig), indicating that these defects are due to loss of these Hox genes rather than other mutations in the genetic background.

We conclude that both *ceh-13* and *nob-1/php-3* are required not only for proper cell positioning, but also for normal division patterns, suggesting a broader role in fate specification, although it remains unclear whether this is controlled directly by the regulation of key fate specifiers. Alternatively, the control of cell identity could be indirect if cell positions are critical for promoting proper local signaling interactions to specify cell fates. Importantly, the changes in division patterns are not consistent with broad homeotic changes in lineage identity. Furthermore, the incomplete penetrance of both cell cycle and cell position defects in *ceh-13* and *nob-1* mutants indicate redundancy in developmental programming that likely contributes to the robustness of *C*. *elegans* embryonic development [33].

## Discussion

Classic work in many species defined the concept of Posterior Dominance, in which posterior Hox genes repress the expression and activity of more anterior Hox genes [1]. Indeed, this principle is evident in later stages of *C*. *elegans* development, where previous work showed that *nob-1* represses *ceh-13* in the posterior ventral nerve cord, consistent with posterior dominance [46]. In contrast, we show that *ceh-13* activates *nob-1/php-3* expression through at least two enhancers during *C*. *elegans* gastrulation. To our knowledge, this is the first example of an anterior Hox gene positively regulating the expression of a posterior Hox gene during development. *ceh-13* and *nob-1/php-3* are also expressed at an earlier phase in development (pre-gastrulation for *ceh-13* and mid-gastrulation for *nob-1/php-3*) than in many other organisms. These observations, along with the lineage defects seen in *ceh-13* and *nob-1* mutants, suggest that the role of *ceh-13* and *nob-1* is to regulate lineage identity, distinct from their later role in positional identity. The role of Hox cofactor genes such as *unc-62*, *ceh-20* and *ceh-40* in early embryonic *nob-1* regulation suggests that these novel functions in lineage specification nonetheless share some common mechanisms with the later roles. The fact that *C*. *elegans* is largely unsegmented may have allowed the co-option of these Hox genes for this distinct role. This emphasizes the striking amount of evolutionary flexibility in the function of the genes of the *C*. *elegans* Hox cluster.

Several studies have highlighted that conserved transcription factors often “moonlight,” with the same factor playing apparently distinct roles in early progenitors and in terminal cell types. For example, the OTX homeodomain TF-encoding gene *ceh-36* and the PITX homeodomain TF-encoding gene *unc-30*, which each specify distinct terminal neuron types, also act redundantly 6–7 cell divisions earlier in the ABp(l/r)p lineages to specify broad features of lineage identity [51,53–55], and *skn-1*, which is required maternally to specify the endomesodermal blastomere EMS at the 4-cell stage is required post-embryonically for oxidative stress resistance and longevity [56,57]. Our work shows that *ceh-13* and *nob-1/php-3* are another example of this phenomenon. Our identification of cell division defects in each mutant demonstrates they also play a role in lineage specification. In addition, we confirmed that each is also required for correct cell position; it is important to note that position defects could also result from incorrect specification of lineage identity or that mispositioning of cells or their progenitors could disrupt additional signals that specify cell fate. Previous work showed that at least one neuron (the PLM touch receptor) is defective in less severe *nob-1* mutants [20]; the failure of PLM neuroblasts to divide in *nob-1/php-3* mutants indicates a function in PLM progenitor lineages. Similarly, *ceh-13* mutants have cell division defects, and *ceh-13* is required for the lineage-specific activity of specific *nob-1* enhancers. Finally, while *elt-1* has a well-defined known role in epidermal fate specification [58], our work shows that it is also required for *nob-1* expression, including in cells that adopt non-epidermal fates. This is consistent with other recent work identifying defects in cell division patterns and the expression of the neurogenic TF-encoding gene *lin-32* in *elt-1* mutants [59]. The ability of developmental transcription factors to “moonlight’ is likely facilitated by complex regulation by multiple enhancers as seen for *nob-1/php-3*, and *ceh-13* [31,32] since enhancer evolution can enable genes to be expressed at different locations and times in development [60,61]. An intriguing question is whether these multiple Hox functions are made possible by the unique nature of the *C*. *elegans* lineage, where positional identity is defined at least in part by lineage history [18,62], or if they are conserved in other species.

While early reporter studies in *C*. *elegans* found that the expression of many genes expressed in terminal cells is well approximated using just the promoter-proximal region [63–65], open chromatin mapping studies have identified many distal regions of open chromatin, many of which can act as enhancers [32,39,40]. Our work reinforces these studies and emphasizes the importance of enhancers in *C*. *elegans* gene regulation. Intriguingly, other genes expressed in lineage-specific patterns around the same time as *nob-1* have similar tendency to have multiple nearby regions of NHR-2::GFP binding and lineage-specific accessible chromatin as measured by ATAC-seq [66], suggesting they may also be regulated by multiple distal enhancers. This could reflect these genes’ need for more complex regulation. The *nob-1/php-3* locus contains at least two or three enhancers responsible for expression in each expression domain during gastrulation, with different enhancers more or less dependent on (or independent of) particular activating TFs. This is reminiscent of so-called “shadow enhancers” identified in other organisms, for which redundancy appears to confer robustness in the face of environmental variability [67–69]. This high level of redundancy also appears to extend to the number of transcription factors regulating each enhancer. Current data implicate at least eight TFs as activators of *nob-1* enhancers including those encoded by the genes *ceh-13*, *elt-1*, *ceh-36*, *unc-30*, the Wnt effector gene *pop-1*, and the Hox co-factor genes *ceh-20*, *ceh-40* and *unc-62* (S9 Fig). In some cases, the same binding motif may be regulated by different factors in different lineages. For example, mutating six GATA motifs in the -3.4 kb enhancer caused the loss of expression in ABp(l/r)ap, potentially due to loss of ELT-1 binding. However, this mutant also lacked reporter fluorescence in Ep, which does not express *elt-1*, but does express genes for other GATA factors with similar binding specificity including *end-1*, *end-3*, and *elt-7* [70]. Similarly, the greater reduction in *nob-1* enhancer reporter fluorescence when CEH-13 motifs are mutated than observed in *ceh-13* mutant animals indicates that other TFs may also bind these sites. The partially penetrant nature of many of the mutant phenotypes further emphasizes the high level of redundancy. In sum, multiple modes of regulatory redundancy could provide a mechanism for the remarkable developmental robustness observed in *C*. *elegans*.

## Methods

### Strain generation and propagation

Worm strains (Table A in S1 Table) were maintained at 21–23°C on OP50 *E*. *coli* on NGM plates. Enhancer reporter strains were generated by injection into RW10029, a GFP histone strain used for lineage tracing. Injection cocktails consisted of reporter DNA construct at 10ng/μL, with 5 ng/μL myo-2p::GFP and 135 ng/μL pBluescript vector and were injected using a Narishige MN-151 micromanipulator with Tritech microinjector system. The *nob-1* promoter deletion strains were generated by injection and compared to a wild-type *nob-1* promoter strain, JIM518, created by injection of pJIM20::nob-1. The *ceh-13* rescuing fosmid transgene, *ujIs153*, was created by bombardment of WRM0622C_C06 [71] as previously described [51]. Other strains were created through crosses using standard approaches. Lethal alleles were maintained as balanced heterozygotes (*ceh-13(sw1))* or rescued by free duplications (*nob-1(ct223)*), with homozygous mutants recognized by characteristic high-penetrance morphology defects. Note, the *ct223* allele disrupts both *nob-1* and *php-3*. Lethal *ceh-36;unc-30* double mutants were maintained as homozygotes rescued by an extra-chromosomal array carrying *ceh-36*::GFP; lack of rescue was scored by absence of GFP in the ABpxpa lineage. Endogenously tagged CRISPR knock-in alleles, *ceh-13(syb2605[ceh-13*::*GFP]) and nob-1(syb2679[nob-1*::*GFP])* were created under contract by SUNY Biotech (Fuzhou, China).

### Molecular biology

Candidate enhancers were amplified with Phusion HF polymerase (New England Biosciences) from either pJIM20::nob-1 or N2 genomic DNA, with overhangs for stitching, which were then either gel or PCR purified (Qiagen). Putative enhancers were attached to a *pes-10* minimal promoter::HIS-24::mCherry::let-858 3’UTR fragment amplified from POPTOP plasmid [72] (Addgene #34848) by using PCR stitching to create an enhancer reporter which was sequence verified and purified with a PureLink PCR purification kit (ThermoFisher) for injection. The *pes-10* minimal promoter drives no consistent early embryonic expression alone, but has previously been shown to facilitate expression driven by different enhancers [72–74]. For the downstream enhancer experiment, the -4.3kb enhancer was cloned immediately downstream of the reporter stop codon. Tested enhancer sequences can be found in Table B in S1 Table. Regions deleted from the -5.3 kb transcriptional reporter match the tested enhancer sequences exactly; deletions were performed using the QuikChange II mutagenesis kit (Agilent). Putative transcription factor binding sites were identified using CIS-BP (cisbp.ccbr.utoronto.ca) [9,49] (Table C-E in S1 Table). DNA fragments with desired mutations were synthesized (Integrated DNA Technologies) and PCR stitched as before.

### Imaging/Lineaging

We acquired confocal images with a Leica TCS SP5, Stellaris or Nikon A1RSi resonance scanning confocal microscope (67 z planes at 0.5 μm spacing and 1.5 minute time spacing, with laser power increasing by 4-fold through the embryo depth to account for attenuation of signal with depth). Embryos from self-fertilized hermaphrodites were mounted in egg buffer/methyl cellulose with 20μm beads as spacers [75] and imaged at 22°C using a stage temperature controller (Brook Industries, Lake Villa, IL). We used StarryNite software to automatically annotate nuclei and trace lineages, and AceTree software to identify and fix any errors from the automated analysis, and quantified reporter expression in each nucleus relative to local background (using the “blot” background correction technique) as previously described [26,27,29,76,77]. In brief, the locally calculated background fluorescence is subtracted from the value measured within the defined nucleus of each cell to reduce false positives from out-of-focus light from nearby expressing cells. Therefore, expressing cells will have positive fluorescence while non-expressing cells can have negative values due to slightly higher autofluorescence in the cytoplasm than in the nucleus. All Cell Data files, which include reporter levels and position information for individual cells at all curated timepoints, are deposited in the Dryad repository: http://doi.org/10.5061/dryad.zs7h44j86 [78].

### Quantitative comparisons

Cell averages of nuclear fluorescence were computed for each cell based on all (typically >20) measurements across its lifetime (Table F in S1 Table). For each control condition, the mean value of each named expressing cell across control embryos was computed and used to determine the fold change for each control and mutant cell with the same name, which were displayed as boxplots using R 3.5.1 (The R Foundation for Statistical Computing). Background-corrected expression values were rounded up to zero if values fell below zero. To determine if changes were significant across the lineage, the sum of expression for each cell in the lineage of interest was calculated for each control and mutant embryo and the two sets of values were compared using a Wilcoxon Ranked Sum test using R. For each condition, at least four control and three mutant embryos were analyzed (exact numbers in Table G in S1 Table). Each embryo analyzed represents an independent biological replicate. To evaluate the enrichment of NHR-2 binding sites, a chi-squared test was used.

### Mutant cell position analysis

Cell position defects were identified as previously [51]. Briefly, we corrected for differences in global division rates (which did not differ dramatically from wild-type) and considered divisions as defective/outliers if they deviated from the wild-type cell cycle length by at least five minutes and had a z-score greater than three (Table H-J in S1 Table). Cell positions (S2–S4 Tables) were corrected for differences in embryo size and rotation and considered defective if they deviated from the expected wild-type position by at least five microns, had a z-score greater than five, and a nearest neighbor score greater than 0.8 (defined empirically based on the distribution of wild-type scores).

### Dryad DOI

http://doi.org/10.5061/dryad.zs7h44j86

## Supporting information

S1 Fig Hox gene are expressed in overlapping patterns. A) Overlap in the expression of endogenously tagged NOB-1::GFP (blue) and PHP-3::GFP (green). Expression is nearly identical (cyan overlap) except *php-3* is expressed more consistently in ABplpapppp. B, C) Early (B) and late (C) overlap of a CEH-13::GFP fosmid translational reporter transgene with a -9kb NOB-1::GFP rescuing translational reporter transgene. The expression patterns are nearly identical to the endogenously tagged alleles except the 9kb NOB-1::GFP reporter has reduced expression in the C lineage (see Fig 2C). D) Expression pattern of a CEH-13::GFP transgene that contains 8.2 kb of upstream sequence plus the first intron. It lacks expression in the MS lineage compared to the other CEH-13 reporters. E) Correlation between average endogenously tagged NOB-1::GFP and PHP-3::GFP nuclear intensity (arbitrary units) for each cell during embryonic development. F) Correlation between average endogenously tagged CEH-13::GFP and NOB-1::GFP nuclear intensity (arbitrary units) for each cell during embryonic development. Note that no cells express high levels of both proteins. G) Correlation between average endogenously tagged CEH-13::GFP and PHP-3::GFP nuclear intensity (arbitrary units) for each cell at each (1.5 minute) time point. Note that no cells express high levels of both proteins.
(TIF)

S2 Fig Some *nob-1* enhancer reporters drive variable ectopic expression. A) Representative examples showing the expression variability for each of the enhancer reporters tested. Colored lines indicate the lineages of interest. Some lineages with no expression are shown as partial trees. B) Brightfield image of a hatched L1 larva carrying the -4.3kb enhancer reporter and showing the “no backend” phenotype characteristic of *nob-1/php-3* mutants. Nearby bead is 20μm in diameter.
(TIF)

S3 Fig *ceh-13* regulates enhancers of *nob-1*. A) Graphs showing the effect of *ceh-13(sw1)* mutation on endogenously tagged NOB-1::GFP, as well as the control values for the *nob-1* enhancer reporters (*sw1* mutant values same as reported in Fig 3). Early cells were considered to be expressing cells born before 200 minutes post-fertilzation in Sulston-adjusted time (i.e. born prior to ~200 cell stage). Number of biological replicates ranges from 3–16. Red * indicates p<0.05 in Wilcoxon Ranked sum test. B) Plots showing average expression vs. time for wild-type CEH-13::GFP (red, n = 4) expression, and endogenously tagged NOB-1::GFP expression in control (dark blue, n = 4) and *ceh-13(sw1)* mutants (light blue, n = 6) in the lineages that generate the specified cells, ABp(l/r)appppp and Abp(l/r)pppaap. C) Graphs showing the effect of *ceh-13(sw1)* mutation on endogenously tagged PHP-3::GFP expression in the specified lineages. Early cells same as in (A). Red * indicates p<0.05 in Wilcoxon Ranked sum test. Trends in ABp(l/r)ap and ABp(l/r)ppp are similar to those for NOB-1::GFP, but do not reach significance likely due to smaller number of mutant embryos analyzed (n = 16 for NOB-1::GFP, n = 4 for PHP-3::GFP). D) Lineage trees showing expression of the wild-type -3.4kb enhancer reporter and a version with mutated HOX sites.
(TIF)

S4 Fig Hox co-factors affect NOB-1::GFP expression in specific lineages. A) Tree showing effects of *unc-*62 RNAi on *nob-1* promoter transcriptional reporter expression in the ABpl lineage. Lineages with loss of expression are underlined. The ABp(l/r)apap lineage showed a loss of reporter fluorescence in 13/14 *unc-62* RNAi lineages examined. B) Trees showing the effect of *unc-*62 RNAi on NOB-1::GFP translational reporter expression, with two examples shown. Lineages affected are underlined. C) Graphs for all embryos tested (n = 8) for the same lineages shown in (B). Red * indicates p<0.05 in Wilcoxon Ranked sum test. D) Effect of *unc-62* RNAi on CEH-13::GFP fosmid reporter in lineages where the two genes are both expressed (n = 4 for control and RNAi). No significant changes were detected.
(TIF)

S5 Fig Full Lineages for *nob-1/php-3* and regulators. Example full lineages for CEH-20::GFP, CEH-40::GFP, UNC-62::GFP, and ELT-1::GFP, all fosmid translational reporters. Full lineages for endogenously tagged CEH-13::GFP, NOB-1::GFP and PHP-3::GFP are also shown. All lineages are shown to at least the 350 cell stage—selected lineages are shown later to identify additional expression or dynamics.
(TIF)

S6 Fig Expression mediated by *nob-1* enhancers requires *elt-1*. A) Fold change values of endogenously tagged NOB-1::GFP and *nob-1* enhancer reporters in control and *elt-1* mutant conditions in the ABp(l/r)ap and Cpap lineages (if expressed in control). Mutant enhancer reporter values are the same as reported in Fig 5. Red * indicates p<0.05 in Wilcoxon Ranked sum test. Number of biological replicates ranges from 4–10. B) Example trees showing expression of endogenously tagged NOB-1::GFP in control and *elt-1* mutant conditions in the specified lineages. Underlined lineages show significant loss of expression (quantified in A). In the C lineage, only underlined cells express ELT-1::GFP fosmid reporter in control embryos. C) Fold change values of endogenously tagged NOB-1::GFP and *nob-1* enhancer reporters in control and *elt-1* mutant conditions in the ABp(l/r)ppp and Ep lineages (if expressed in control) where ELT-1::GFP is not expressed. Red * indicates p<0.05 in Wilcoxon Ranked sum test. D) Expression of the -3.4kb enhancer reporter and a version from which all GATA sites have been mutagenized in the ABp, E and C lineages. Note: the mutagenesis also disrupted one *ceh-20/40* predicted site, two *nob-1* predicted sites, and one *pop-1* predicted site, as these were fully overlapping with GATA sites.
(TIF)

S7 Fig *ceh-36* and *unc-30* contribute to *nob-1* expression. A) Wild-type and *ceh-36(ok795);unc-30(ok613)* mutant embryos expressing the NOB-1::GFP reporter transgene. Expression is shown in the ABp lineage at the ~200 cell stage. Note: *ceh-36* and *unc-30* are not expressed in the ABp(l/r)ap lineages (pink underline). B) Boxplot showing the ratio of expression in the ABp(l/r)ap lineage to the ABp(l/r)ppp lineage for wild-type and *ceh-36(ok795);unc-30(ok613)* mutant at the 350 cell stage for at least 4 embryos. Red * indicates p<0.05 in Wilcoxon Ranked sum test.
(TIF)

S8 Fig Defective positions of specific cells in *nob-1* mutants. A) The position of cells that normally express *nob-1* are highlighted in the context of the whole embryo at ~320 minutes (~570 cells, early morphogenesis). As compared to the wild-type average (left), the *nob-1(ct223)* mutant (right) cells are disorganized and displaced anteriorly, particularly ABp(l/r)ppp and some ABp(l/r)app cells (ABp(l/r)appp). B) The positions of the SAB neuron and neuroblasts (red) and DA motorneurons (blue) are shown relative to the intestine (green) in wild-type (left) and *ceh-13(sw1)* mutant (right), showing the anterior displacement of these cells. C) The positions of the neuroblasts that fail to divide the *nob-1(ct223)* mutant as compared to control (left), showing that these are dramatically mispositioned. D) Cell division defects in *nob-1(ct351)* and *ceh-13(ok737)* mutants. Defects highlighted are the same as shown in Fig 6J and 6K, and fraction of observed cells from each genotype that have each defect are noted.
(TIF)

S9 Fig Regulation of *nob-1/php-3* at the lineage and enhancer levels. A) Diagram summarizing the transcriptional regulation of *nob-1/php-3* expression in the specific lineages noted. Asterisk indicates indirect regulation. Note: although *nob-1/php-3* are expressed in other lineages in C, only Cpap was possible to analyze with the transgenic reporters examined. Regulation by *pop-1/sys-1* from Zacharias *et al*., 2015 [24]. B) Diagram summarizing enhancer-level transcriptional regulation of *nob-1/php-3* by the indicated factors. Specific enhancers regulated by *ceh-20/ceh-40*, *ceh-36/unc-30*, *pop-1/sys-1* and *unc-62* were not defined, but their activity can be localized to the regions marked by brackets. Based on our results, additional *cis*-regulatory elements likely exist within the blue, green and yellow regions (yellow encompasses the rest of the genome).
(TIF)

S1 Table Table A: Strain list. Table B: Tested enhancer sequences. Tables C-E: Motifs found in *nob-1* enhancers -3.4kb, -4.3kb, -8.3kb. Table F: Cell averaged reporter values for all embryos analyzed. Table G: Number of embryos analyzed for each genotype/condition. Table H-J: Cell division times for wild-type, *ceh-13(sw1)*, and *nob-1(ct223)* embryos.
(XLSX)

S2 Table Cell position values for control embryos. (ZIP)

S3 Table Cell position values for *ceh-13(sw1)* embryos. (CSV)

S4 Table Cell position values for *nob-1(ct223)* embryos. (CSV)
