# Supplementary figures and images for: The anterior Hox gene ceh-13 and elt-1/GATA activate the posterior Hox genes nob-1 and php-3 to specify posterior lineages in the C. elegans embryo
Source: PLoS Genet. 2022 May 2;18(5):e1010187. doi: 10.1371/journal.pgen.1010187 (PMC9098060; doi:10.1371/journal.pgen.1010187)

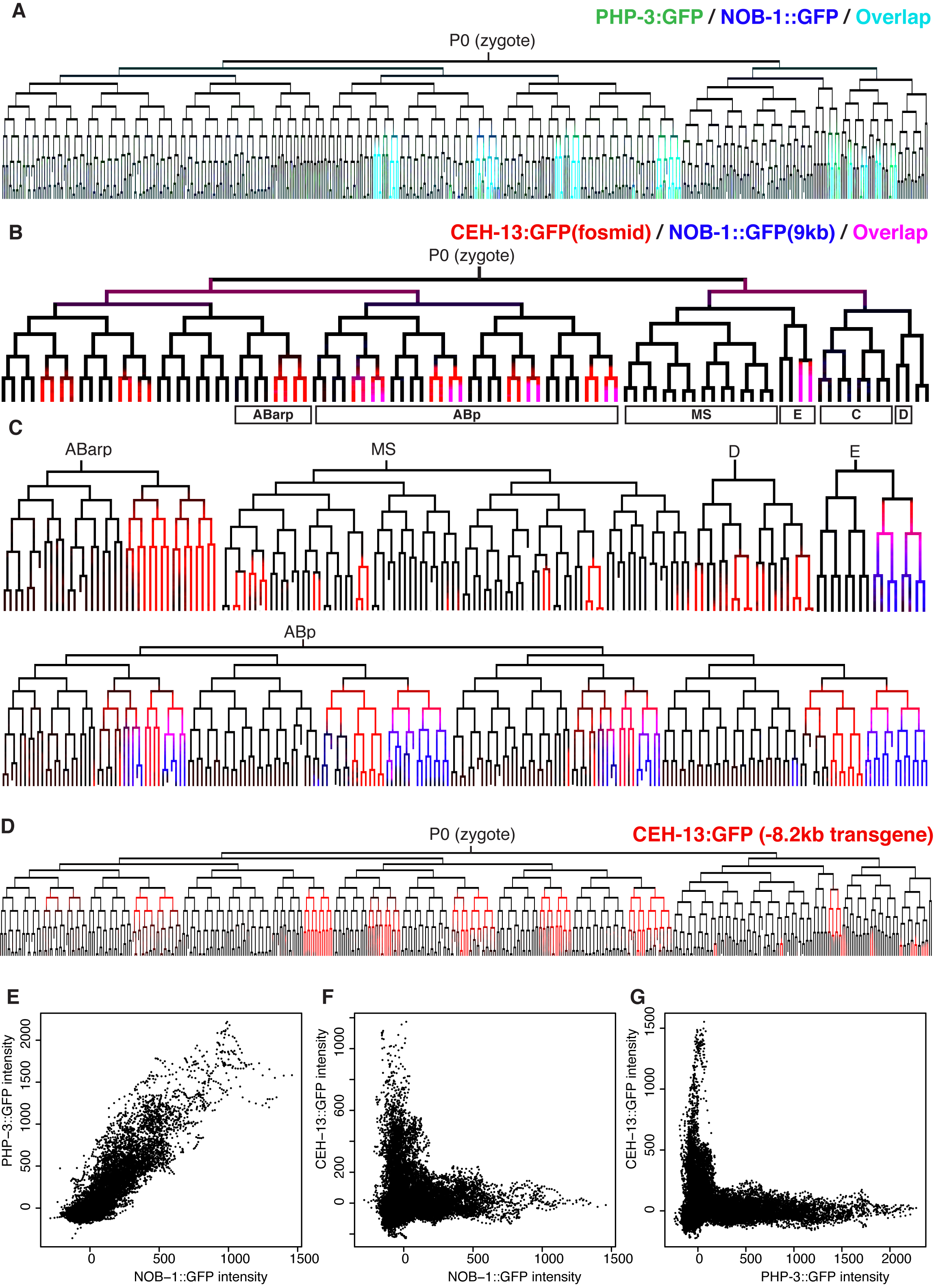

Supplement: S1 Fig — A) Overlap in the expression of endogenously tagged NOB-1::GFP (blue) and PHP-3::GFP (green). Expression is nearly identical (cyan overlap) except php-3 is expressed more consistently in ABplpapppp. B, C) Early (B) and late (C) overlap of a CEH-13::GFP fosmid translational reporter transgene with a -9kb NOB-1::GFP rescuing translational reporter transgene. The expression patterns are nearly identical to the endogenously tagged alleles except the 9kb NOB-1::GFP reporter has reduced expression in the C lineage (see Fig 2C). D) Expression pattern of a CEH-13::GFP transgene that contains 8.2 kb of upstream sequence plus the first intron. It lacks expression in the MS lineage compared to the other CEH-13 reporters. E) Correlation between average endogenously tagged NOB-1::GFP and PHP-3::GFP nuclear intensity (arbitrary units) for each cell during embryonic development. F) Correlation between average endogenously tagged CEH-13::GFP and NOB-1::GFP nuclear intensity (arbitrary units) for each cell during embryonic development. Note that no cells express high levels of both proteins. G) Correlation between average endogenously tagged CEH-13::GFP and PHP-3::GFP nuclear intensity (arbitrary units) for each cell at each (1.5 minute) time point. Note that no cells express high levels of both proteins. (TIF) [file pgen.1010187.s001.tif]

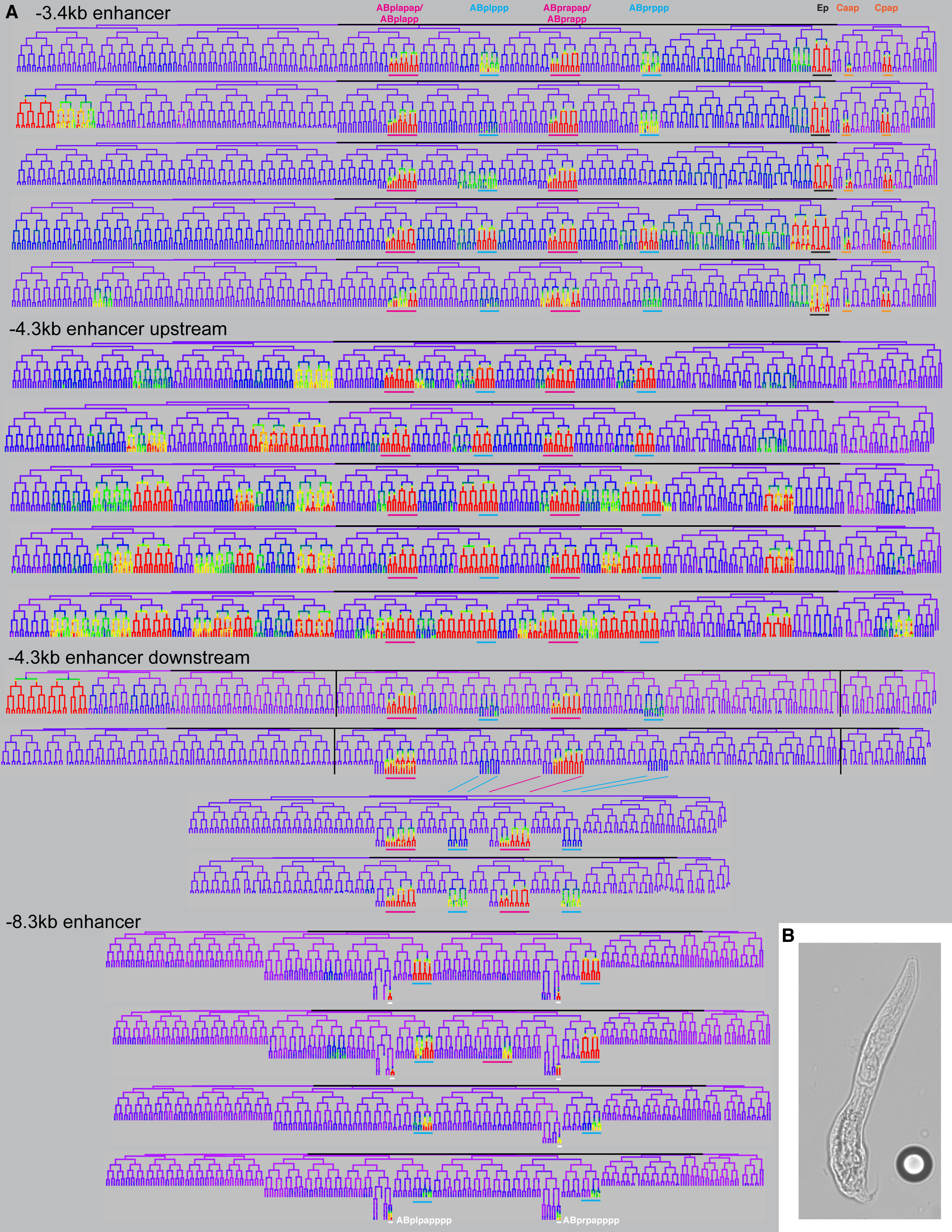

Supplement: S2 Fig — A) Representative examples showing the expression variability for each of the enhancer reporters tested. Colored lines indicate the lineages of interest. Some lineages with no expression are shown as partial trees. B) Brightfield image of a hatched L1 larva carrying the -4.3kb enhancer reporter and showing the “no backend” phenotype characteristic of nob-1/php-3 mutants. Nearby bead is 20μm in diameter. (TIF) [file pgen.1010187.s002.tif]

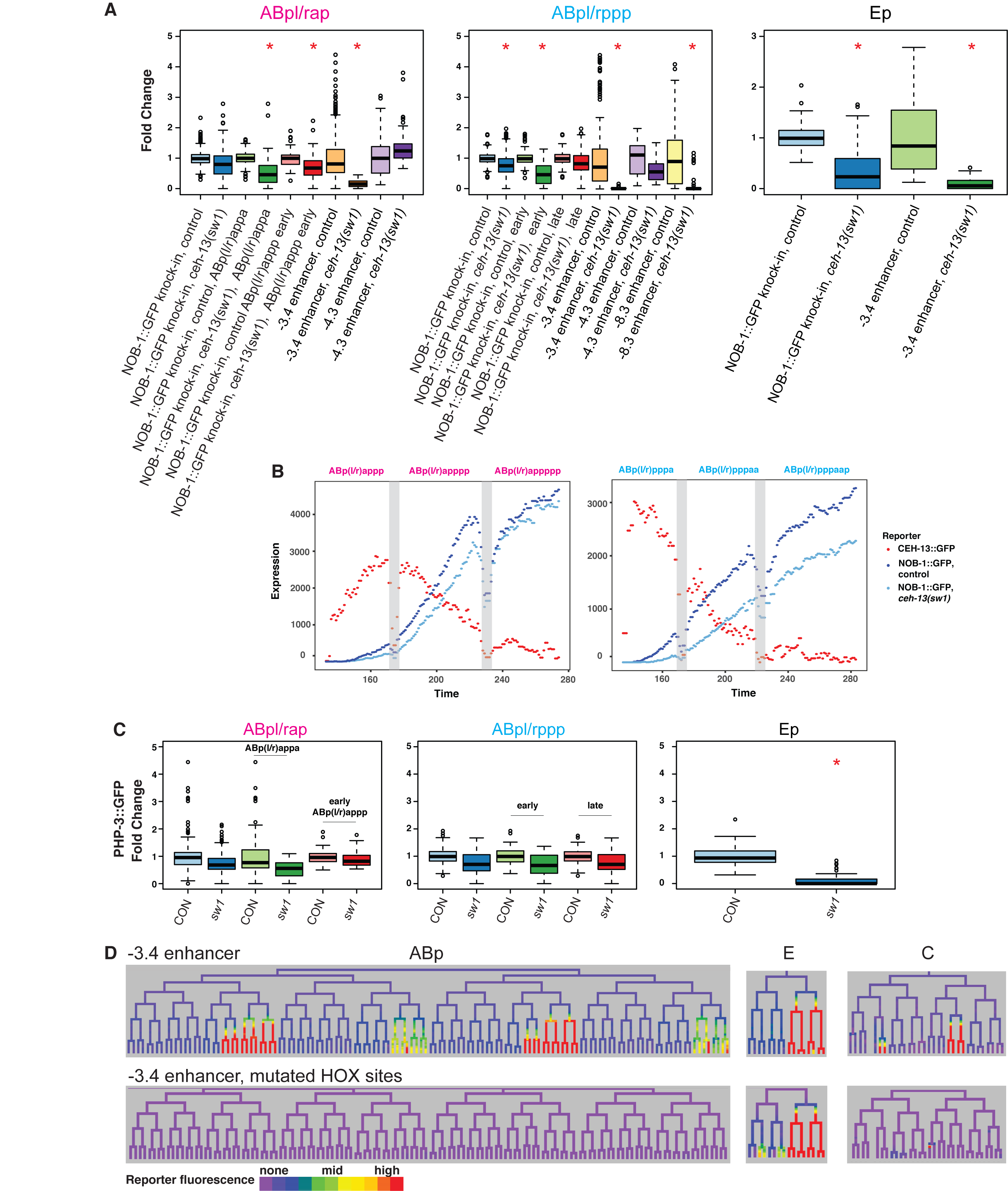

Supplement: S3 Fig — A) Graphs showing the effect of ceh-13(sw1) mutation on endogenously tagged NOB-1::GFP, as well as the control values for the nob-1 enhancer reporters (sw1 mutant values same as reported in Fig 3). Early cells were considered to be expressing cells born before 200 minutes post-fertilzation in Sulston-adjusted time (i.e. born prior to ~200 cell stage). Number of biological replicates ranges from 3–16. Red * indicates p<0.05 in Wilcoxon Ranked sum test. B) Plots showing average expression vs. time for wild-type CEH-13::GFP (red, n = 4) expression, and endogenously tagged NOB-1::GFP expression in control (dark blue, n = 4) and ceh-13(sw1) mutants (light blue, n = 6) in the lineages that generate the specified cells, ABp(l/r)appppp and Abp(l/r)pppaap. C) Graphs showing the effect of ceh-13(sw1) mutation on endogenously tagged PHP-3::GFP expression in the specified lineages. Early cells same as in (A). Red * indicates p<0.05 in Wilcoxon Ranked sum test. Trends in ABp(l/r)ap and ABp(l/r)ppp are similar to those for NOB-1::GFP, but do not reach significance likely due to smaller number of mutant embryos analyzed (n = 16 for NOB-1::GFP, n = 4 for PHP-3::GFP). D) Lineage trees showing expression of the wild-type -3.4kb enhancer reporter and a version with mutated HOX sites. (TIF) [file pgen.1010187.s003.tif]

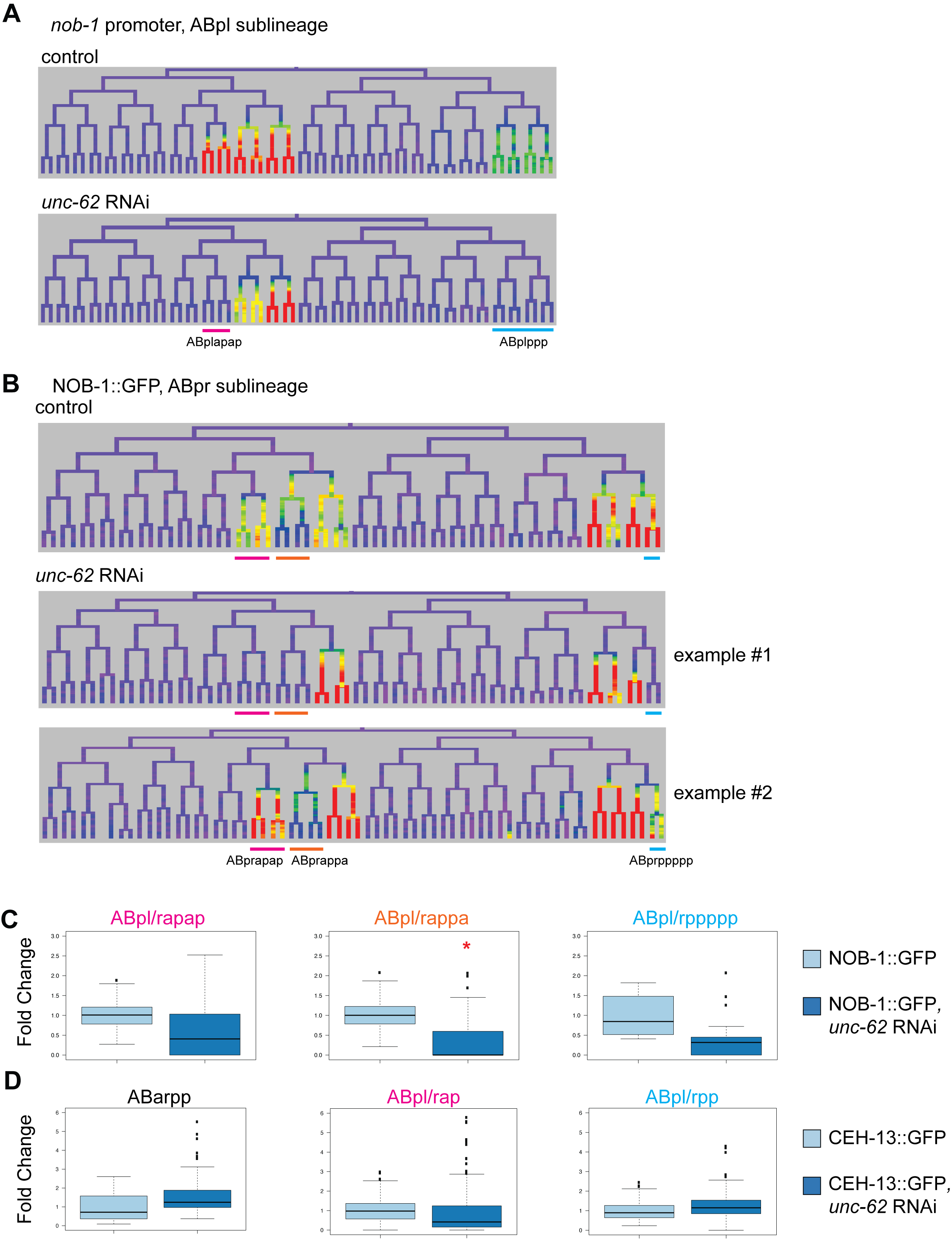

Supplement: S4 Fig — A) Tree showing effects of unc-62 RNAi on nob-1 promoter transcriptional reporter expression in the ABpl lineage. Lineages with loss of expression are underlined. The ABp(l/r)apap lineage showed a loss of reporter fluorescence in 13/14 unc-62 RNAi lineages examined. B) Trees showing the effect of unc-62 RNAi on NOB-1::GFP translational reporter expression, with two examples shown. Lineages affected are underlined. C) Graphs for all embryos tested (n = 8) for the same lineages shown in (B). Red * indicates p<0.05 in Wilcoxon Ranked sum test. D) Effect of unc-62 RNAi on CEH-13::GFP fosmid reporter in lineages where the two genes are both expressed (n = 4 for control and RNAi). No significant changes were detected. (TIF) [file pgen.1010187.s004.tif]

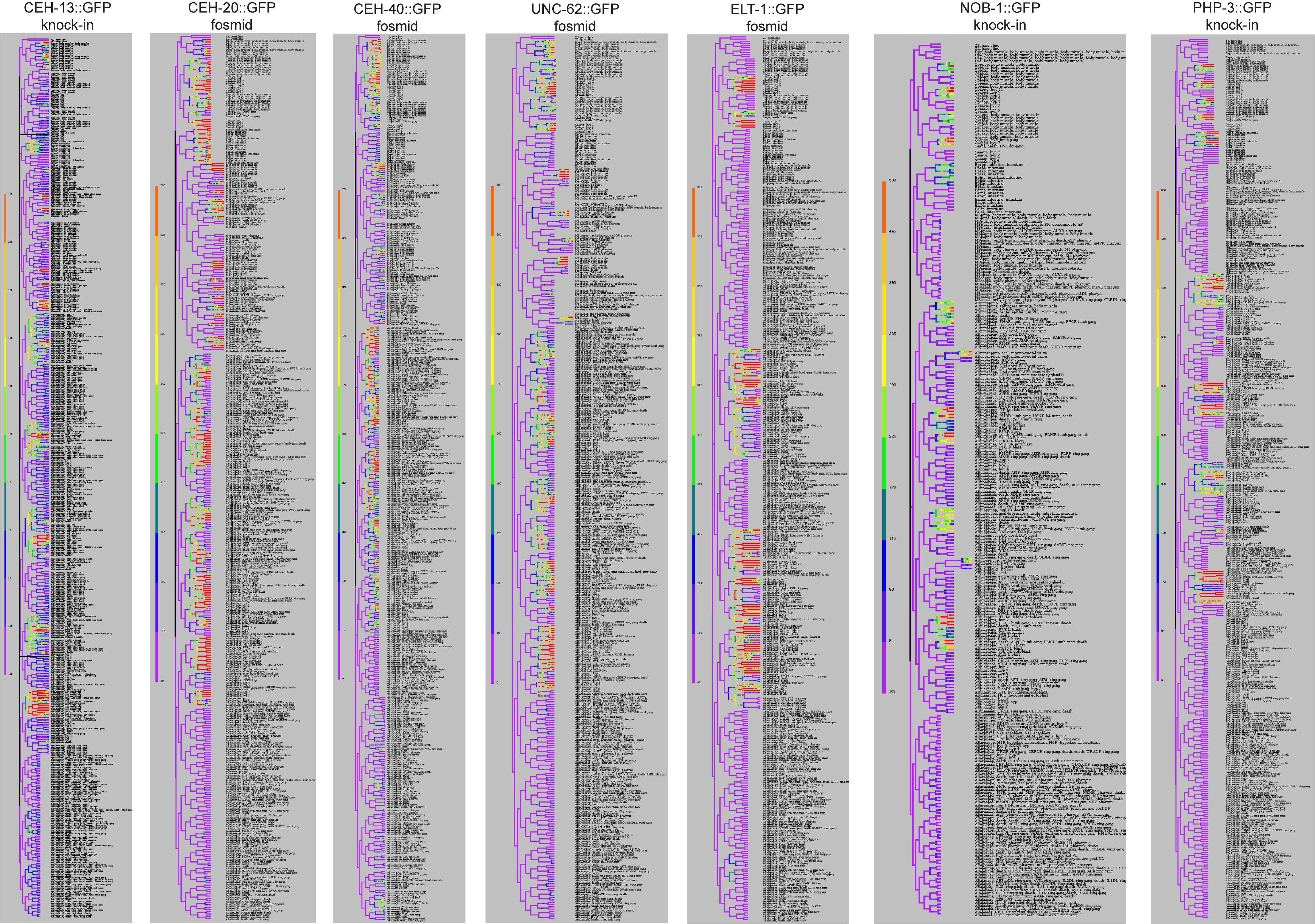

Supplement: S5 Fig — Example full lineages for CEH-20::GFP, CEH-40::GFP, UNC-62::GFP, and ELT-1::GFP, all fosmid translational reporters. Full lineages for endogenously tagged CEH-13::GFP, NOB-1::GFP and PHP-3::GFP are also shown. All lineages are shown to at least the 350 cell stage—selected lineages are shown later to identify additional expression or dynamics. (TIF) [file pgen.1010187.s005.tif]

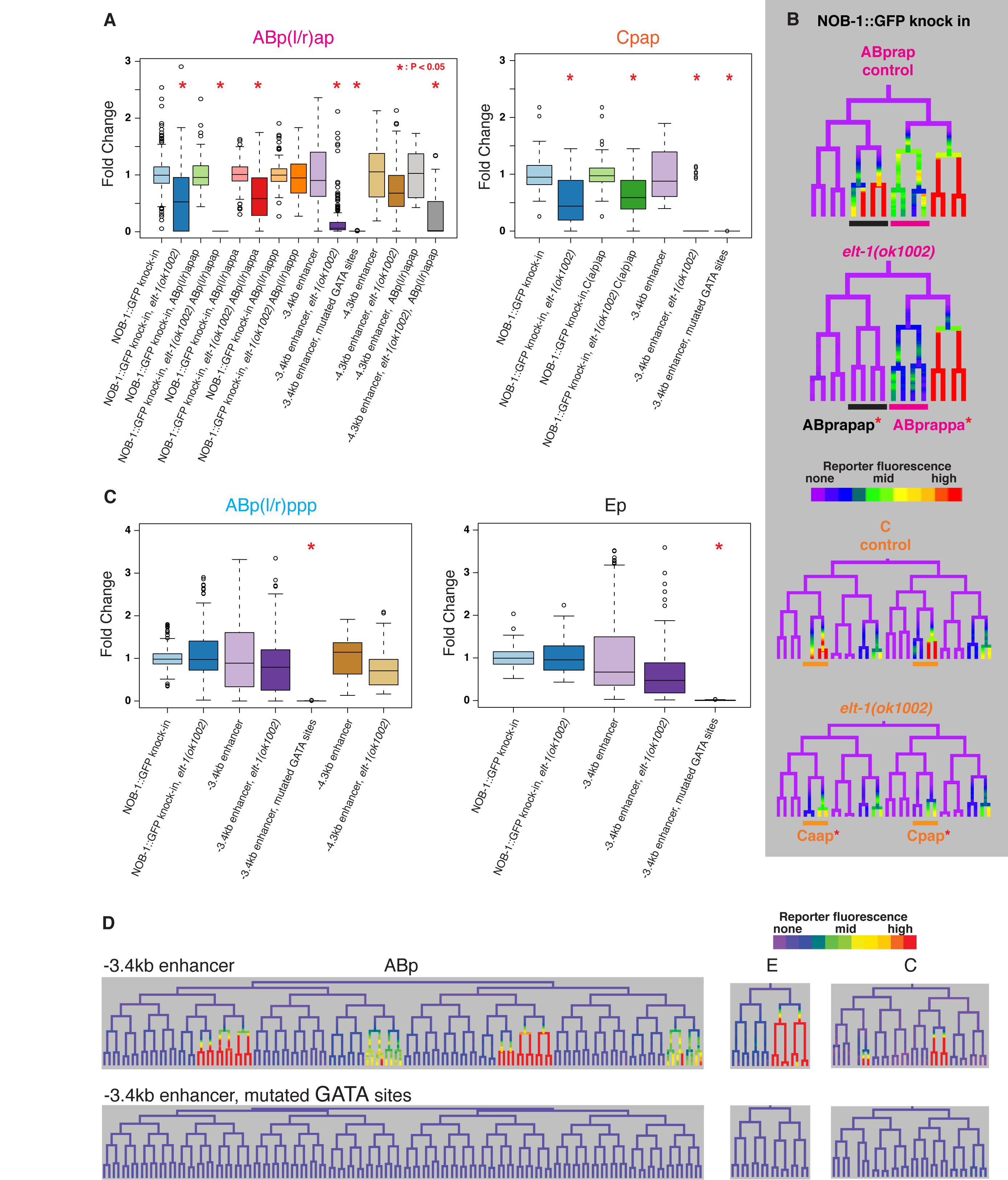

Supplement: S6 Fig — A) Fold change values of endogenously tagged NOB-1::GFP and nob-1 enhancer reporters in control and elt-1 mutant conditions in the ABp(l/r)ap and Cpap lineages (if expressed in control). Mutant enhancer reporter values are the same as reported in Fig 5. Red * indicates p<0.05 in Wilcoxon Ranked sum test. Number of biological replicates ranges from 4–10. B) Example trees showing expression of endogenously tagged NOB-1::GFP in control and elt-1 mutant conditions in the specified lineages. Underlined lineages show significant loss of expression (quantified in A). In the C lineage, only underlined cells express ELT-1::GFP fosmid reporter in control embryos. C) Fold change values of endogenously tagged NOB-1::GFP and nob-1 enhancer reporters in control and elt-1 mutant conditions in the ABp(l/r)ppp and Ep lineages (if expressed in control) where ELT-1::GFP is not expressed. Red * indicates p<0.05 in Wilcoxon Ranked sum test. D) Expression of the -3.4kb enhancer reporter and a version from which all GATA sites have been mutagenized in the ABp, E and C lineages. Note: the mutagenesis also disrupted one ceh-20/40 predicted site, two nob-1 predicted sites, and one pop-1 predicted site, as these were fully overlapping with GATA sites. (TIF) [file pgen.1010187.s006.tif]

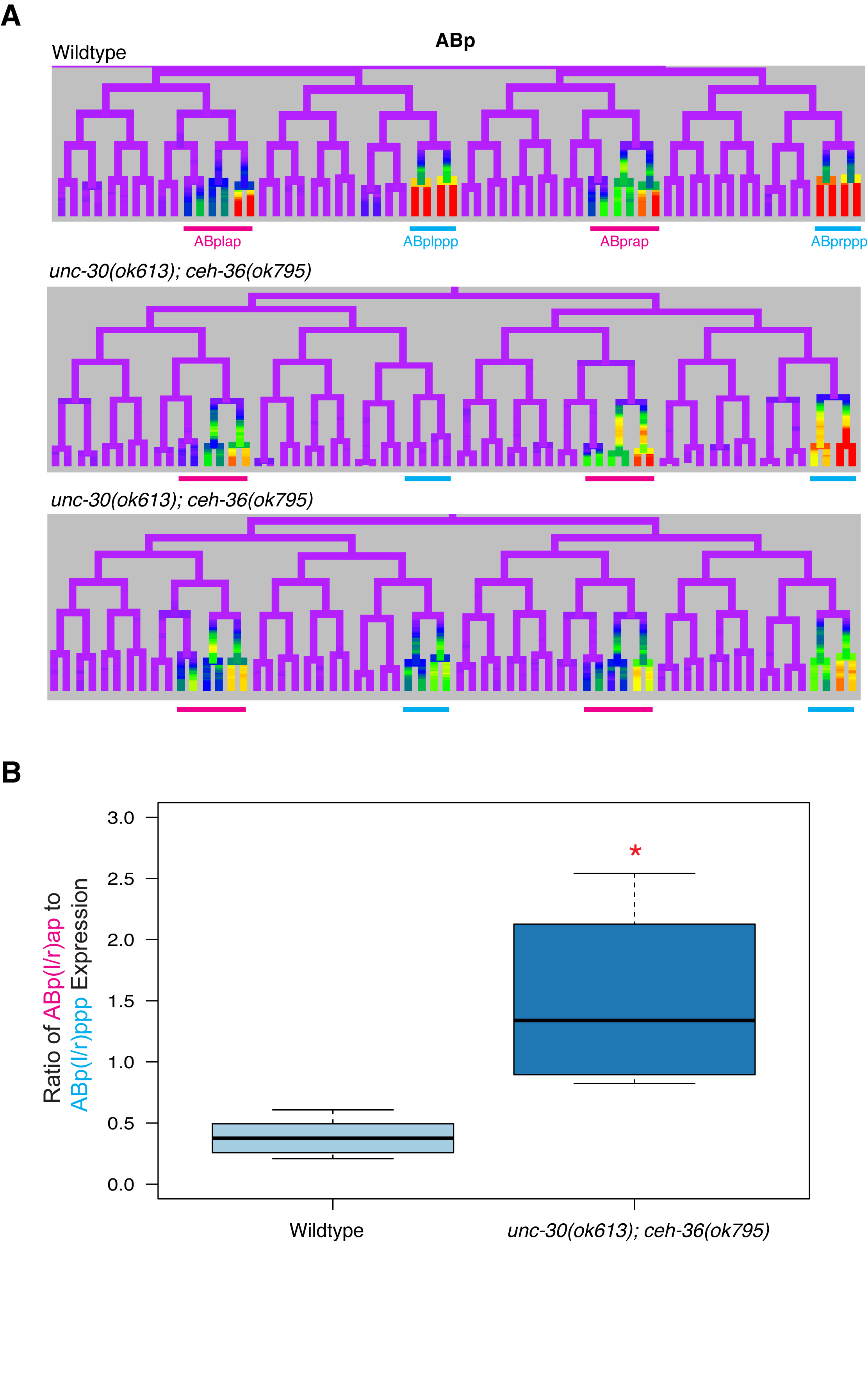

Supplement: S7 Fig — A) Wild-type and ceh-36(ok795);unc-30(ok613) mutant embryos expressing the NOB-1::GFP reporter transgene. Expression is shown in the ABp lineage at the ~200 cell stage. Note: ceh-36 and unc-30 are not expressed in the ABp(l/r)ap lineages (pink underline). B) Boxplot showing the ratio of expression in the ABp(l/r)ap lineage to the ABp(l/r)ppp lineage for wild-type and ceh-36(ok795);unc-30(ok613) mutant at the 350 cell stage for at least 4 embryos. Red * indicates p<0.05 in Wilcoxon Ranked sum test. (TIF) [file pgen.1010187.s007.tif]

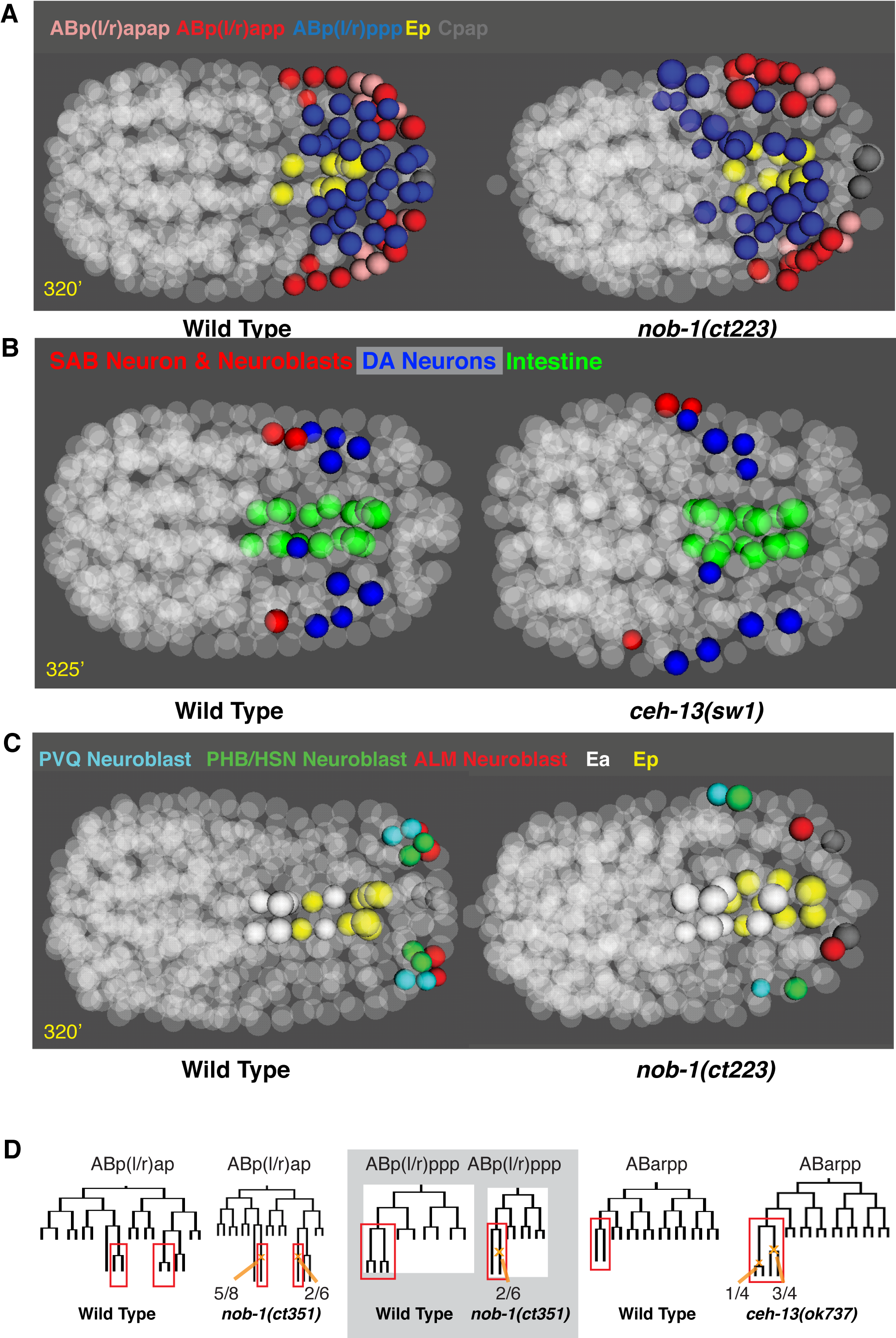

Supplement: S8 Fig — A) The position of cells that normally express nob-1 are highlighted in the context of the whole embryo at ~320 minutes (~570 cells, early morphogenesis). As compared to the wild-type average (left), the nob-1(ct223) mutant (right) cells are disorganized and displaced anteriorly, particularly ABp(l/r)ppp and some ABp(l/r)app cells (ABp(l/r)appp). B) The positions of the SAB neuron and neuroblasts (red) and DA motorneurons (blue) are shown relative to the intestine (green) in wild-type (left) and ceh-13(sw1) mutant (right), showing the anterior displacement of these cells. C) The positions of the neuroblasts that fail to divide the nob-1(ct223) mutant as compared to control (left), showing that these are dramatically mispositioned. D) Cell division defects in nob-1(ct351) and ceh-13(ok737) mutants. Defects highlighted are the same as shown in Fig 6J and 6K, and fraction of observed cells from each genotype that have each defect are noted. (TIF) [file pgen.1010187.s008.tif]

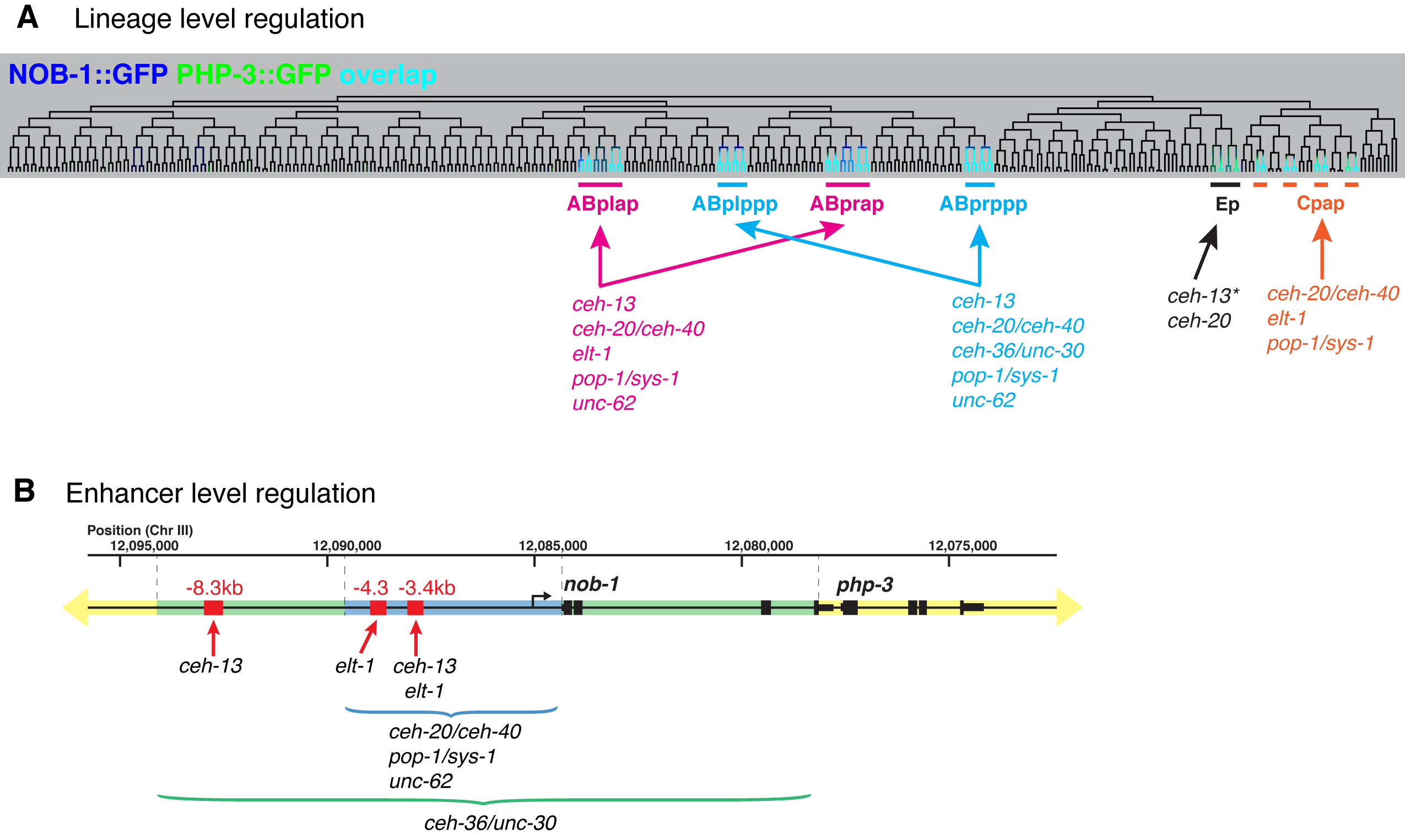

Supplement: S9 Fig — A) Diagram summarizing the transcriptional regulation of nob-1/php-3 expression in the specific lineages noted. Asterisk indicates indirect regulation. Note: although nob-1/php-3 are expressed in other lineages in C, only Cpap was possible to analyze with the transgenic reporters examined. Regulation by pop-1/sys-1 from Zacharias et al., 2015 [24]. B) Diagram summarizing enhancer-level transcriptional regulation of nob-1/php-3 by the indicated factors. Specific enhancers regulated by ceh-20/ceh-40, ceh-36/unc-30, pop-1/sys-1 and unc-62 were not defined, but their activity can be localized to the regions marked by brackets. Based on our results, additional cis-regulatory elements likely exist within the blue, green and yellow regions (yellow encompasses the rest of the genome). (TIF) [file pgen.1010187.s009.tif]
